# Wheat improvement through advances in single nucleotide polymorphism (SNP) detection and genotyping with a special emphasis on rust resistance

**DOI:** 10.1007/s00122-024-04730-w

**Published:** 2024-09-16

**Authors:** Subramaniam Geethanjali, Palchamy Kadirvel, Sambasivam Periyannan

**Affiliations:** 1 Centre for Plant Molecular Biology and Biotechnology, Tamil Nadu Agricultural University, Coimbatore, Tamil Nadu 641003 India; 2 Centre for Crop Health, University of Southern Queensland, Toowoomba, Queensland 4350 Australia; 3 Crop Improvement Section, Indian Council of Agricultural Research-Indian Institute of Oilseeds Research, Hyderabad, Telangana 500030 India; 4 School of Agriculture and Environmental Science, University of Southern Queensland, Toowoomba, Queensland 4350 Australia

## Abstract

**Key message:**

Single nucleotide polymorphism (SNP) markers in wheat and their prospects in breeding with special reference to rust resistance.

**Abstract:**

Single nucleotide polymorphism (SNP)-based markers are increasingly gaining momentum for screening and utilizing vital agronomic traits in wheat. To date, more than 260 million SNPs have been detected in modern cultivars and landraces of wheat. This rapid SNP discovery was made possible through the release of near-complete reference and pan-genome assemblies of wheat and its wild relatives, coupled with whole genome sequencing (WGS) of thousands of wheat accessions. Further, genotyping customized SNP sites were facilitated by a series of arrays (9 to 820Ks), a cost effective substitute WGS. Lately, germplasm-specific SNP arrays have been introduced to characterize novel traits and detect closely linked SNPs for marker-assisted breeding. Subsequently, the kompetitive allele-specific PCR (KASP) assay was introduced for rapid and large-scale screening of specific SNP markers. Moreover, with the advances and reduction in sequencing costs, ample opportunities arise for generating SNPs artificially through mutations and in combination with next-generation sequencing and comparative genomic analyses. In this review, we provide historical developments and prospects of SNP markers in wheat breeding with special reference to rust resistance where over 50 genetic loci have been characterized through SNP markers. Rust resistance is one of the most essential traits for wheat breeding as new strains of the *Puccinia* fungus, responsible for rust diseases, evolve frequently and globally.

**Supplementary Information:**

The online version contains supplementary material available at 10.1007/s00122-024-04730-w.

## Introduction

Wheat is one of the most important staple food crops, next to rice and maize (Erenstein et al. 2022). In 2022, the wheat production was 808 million tonnes harvested from a land area of 219 million hectares (FAO 2022). Asia contributes 44% of the world's wheat production, followed by Europe (34%) and America (15%). China, India, Russia, the United States of America (USA) and France are the primary producers, contributing more than 50% of the global wheat production (Erenstein et al. 2022). By 2050, the human population is expected to reach 9.7 billion, which implies the necessity for 132 million tonnes of additional wheat by 2050. Hence, there is a need for continued improvement in wheat yield in addition to the challenges posed by biotic and abiotic stresses (Erenstein et al. 2022). Diseases such as rust caused by the fungus *Puccinia* significantly threaten global wheat production. Rust in wheat is of three types: leaf (caused by *P. triticina*), stripe (*P. striiformis* f. sp. *tritici*) and stem (*P. graminis* f. sp. *tritici*) rusts, each causing severe yield losses during peak epidemic.

Globally, rusts in wheat are controlled routinely through fungicide applications and genetic resistance, the innate ability to resist pathogen infection. The latter strategy is essential as they are durable, cost-effective and eco-friendly. Genetic resistance to rust is of two major types: all-stage resistance (ASR) and adult plant resistance (APR). ASR, also known as seedling resistance, is active at all growth stages from seedling emergence. ASR is race-specific, qualitative and controlled by major genes. In contrast, APR is quantitative, controlled by minor genes with cumulative effects. APR is further grouped as early-stage (APR I) and late-stage (APR II) APRs. APR I refers to resistance observed from the fourth leaf stage and is often race-specific, effective against selected races of the pathogen. In contrast, resistance in APR II is evident only at the flag leaf stage and may work against a particular or multiple pathogen(s) but is non-race-specific primarily (Norman et al. 2023).

More than 200 resistance (*R*) genes have been identified against rust, mainly by screening germplasm of cultivated wheat and its wild relatives (Kumar et al. 2022; McIntosh et al. 2022; Norman et al. 2023). To date, 920 quantitative trait loci (QTL) for rust resistance, with 406, 296 and 180 QTLs for stripe, leaf and stem rust resistance, respectively, were mapped in wheat (Tong et al. 2024). The number increases steadily as new pathotypes of *Puccinia* with virulence to widely deployed resistance genes evolve frequently; hence, replacing defeated genes is a routine task for wheat breeders. Further increase in gene discovery is due to the rapid ability to characterize new resistance genes by using molecular markers and comparative genomics. Historically, the intensive use of markers for wheat breeding began in 1990s with the use of hybridization-based restriction fragment length polymorphism (RFLP) markers (reviewed by Rasheed and Xia 2019). Here, DNA fragments of varying lengths produced due to differences at the restriction enzyme sites were distinguished using short DNA sequence probes. The RFLP markers were replaced with the advent of PCR-based markers such as randomly amplified polymorphic DNA (RAPD), amplified fragment length polymorphism (AFLP) and simple sequence repeats (SSR) that involve DNA sequence amplification using random or sequence-specific primers. Among them, SSRs have been the markers of choice for breeders owing to their locus specificity, codominant nature, high level of polymorphism and reproducibility (Rasheed and Xia 2019). Later, with next-generation sequencing (NGS), variation at a single or few nucleotide levels, called single nucleotide polymorphism (SNP) and insertions/deletions (InDels) gained popularity as markers. Naturally, SNPs arise through point mutations leading to transition (changes within purine or pyrimidine) and transversion (interchange between purine and pyrimidine) while InDels arise due to insertion or deletion of few nucleotides or short DNA fragments. However, due to cost and limitations for high-throughput genotyping, SNPs have become popular than InDels in addition to their abundance in any given genome (Gupta et al. 1994; Brookes 1999; Rasheed and Xia 2019; Song et al. 2023).

## Mapping of rust resistance genes using SNP markers

SNPs are used extensively in constructing genetic linkage maps for major genes and QTLs linked to rust resistance. It involves the positioning and identification of markers linked with resistance and is based on marker-trait association analysis performed using mapping populations. Second filial generation (F_2_), recombinant inbred lines (RIL), doubled haploid lines (DHL), backcross inbred lines (BIL) and near-isogenic lines (NIL) are the commonly used mapping populations for mapping rust resistance loci. So far, 296 genetic loci inclusive of 50 designated *R* genes for leaf rust, 406 loci with 39 designated *R* genes for stripe rust and 180 loci with 65 designated *R* genes for stem rust have been mapped (Tong et al. 2024). Among these, 35 leaf rust resistance (*Lr*), 30 stem rust resistance (*Sr*)*,* 17 yellow/stripe rust resistance (*Yr*) genes and numerous QTLs were mapped using SNPs and one of the above biparental mapping populations (Supplementary Table 1a, 1b, 1c and 2).

Genome-wide association study (GWAS) or linkage disequilibrium (LD) mapping is commonly used for identifying novel QTLs, particularly from germplasm or multi-parent advanced generation inter-cross (MAGIC) and nested association mapping (NAM)-based segregating populations (Supplementary Table 3). Moreover, combining linkage and association mapping approaches remains efficient for detecting novel QTLs (Zhou et al. 2022).

Recently, haplotype analysis has emerged as a practical approach to gene mapping (Bhat et al. 2021). A SNP haplotype refers to two or more polymorphic SNPs that inherit together and have strong LD between each other. The haplotype markers are more accurate than the single SNPs for trait prediction and are exploited to identify wheat rust resistance genes (Athiyannan et al. 2022a, 2022b; Bouvet et al. 2022).

Further, identification of the underlying candidate genes for *Lr21*, *Lr42*, *Sr13*, *Sr21*, *Sr22b*, *Sr60, Sr62, Yr5* and *Yr28* ASRs and two pleiotropic (*Lr34*/*Yr18*/*Sr57* and *Lr67*/*Yr46*/*Sr55*) APR II genes has led to the identification of diagnostic SNP markers (Table 1).

**Table 1:** Diagnostic SNP markers for cloned rust resistance genes

Resistance Genes	Resistance type	Source species	Chromosome	Method of cloning	Resistance gene type	Diagnostic SNP markers	Assay type	References
*Lr9*	ASR	*Aegilops umbellulata*	6U	MutIsoSeq	Tandem kinase	TA10438-F, *Lr9*-F, R	KASP	Wang et al. (2023)
*Lr21*	ASR	*Ae.* *tauschii*	1D	Map based	NLR	KSUD14-STS *Lr21*_GQ504819_1346_C/T	STS KASP	Huang et al. (2003), Neelam et al. (2013)
*Lr42*	ASR	*Ae. tauschii*	1D	Map based	NLR	pC43	KASP	Lin et al. (2022)
*Sr13*	ASR	*Triticum turgidum* ssp. *turgidum*	6A	Map based	NLR	T2200C-*Sr13*F/R	CAPS	Zhang et al. (2017)
						rwgsnp7, rwgsnp37, rwgsnp38, rwgsnp39, rwgsnp40	STARP	Sharma et al. (2019, Gill et al. (2021)
						KaspSr13	KASP	Sharma et al. (2019)
*Sr21*	ASR	*T. monococcum*	2A	Map based	NLR	SNPC1228W	CAPS	Chen et al. (2018)
*Sr22b*	ASR	*T. monococcum*	7A	Map based	NLR	TM5TF2R2 pkw4974	InDels CAPS	Luo et al. (2022)
*Sr60*	ASR	*T. monococcum*	5A	Map based	Tandem kinase	*Sr60*F2/R2, DK722976F5R5	CAPS	Chen et al. (2020)
*Sr62*	ASR	*Ae. sharonensis*	1S^sh^	MutRNASeq	Tandem kinase	S741_KASP-7 C03246_CAPS	KASP CAPS	Yu et al. 2022)
*Yr5*	ASR	*T. spelta album*	2B	MutRenSeq	NLR	*Yr5*_KASP	KASP	Marchal et al. (2018)
*Yr28* (*YrAs2388*)	ASR	*Ae. tauschii*	4D	Map based	NLR	HTM3g	CAPS	Zhang et al. (2019a)
						KASP-E5 KASP-E6	KASP	Hu et al. (2021)
*Yr46*/*Lr67*/*Sr55*	APR II	*T. aestivum*	4D	Map based	Hexose transporter	TM4, TM10	KASP	Moore et al. (2015)
*Yr18*/*Lr34*/*Sr57*	APR II	*T. aestivum*	7D	Map based	ATP binding cassette transporter	cssfr6	CAPS	Lagudah et al. (2009)

*ASR* Adult stage resistance, *APR* adult plant resistance, *MutRenSeq* mutagenesis resistance gene enrichment and sequencing, *NLR* nucleotide binding site leucine-rich repeats, *STS* sequence tagged sites, *KASP* kompetitive allele-specific PCR, *CAPS* cleaved amplified polymorphic sequences, *STARP* semi-thermal asymmetric reverse PCR, *InDels* insertions/deletions

## SNP identification in pre-wheat genome sequencing era

**Fig. 1:**
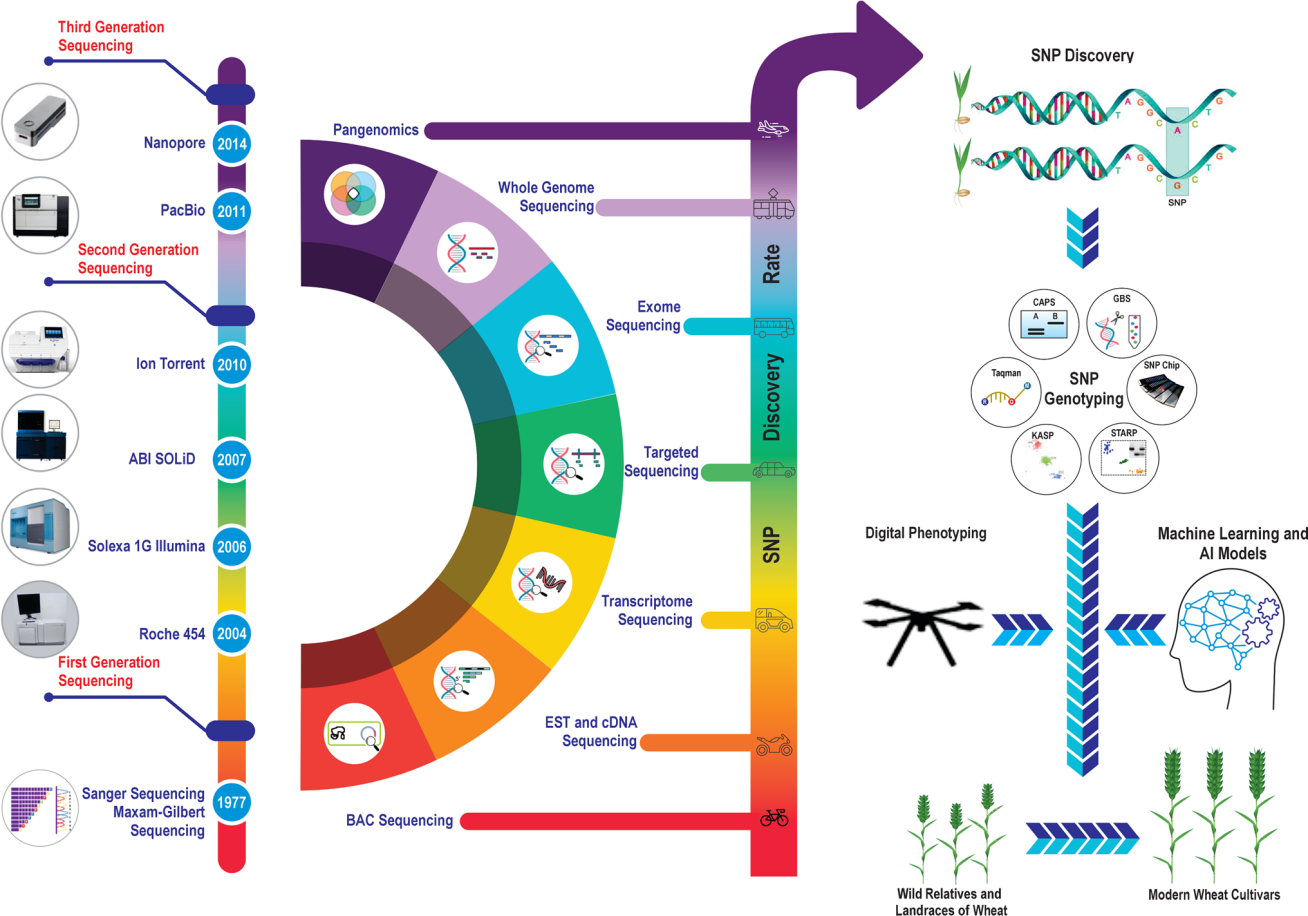
Illustration of technological advancements for SNP discovery and genotyping and their influence on crop improvement. The left-hand panel illustrates the growth in sequencing techniques, while the middle panel on the various resources used for SNP discovery and the right panel outlines the role of SNP in crop improvement

Prior to the sequencing of genomes of crops and their wild species, SNP identification relied heavily on sequence information from RFLP probes and expressed sequence tags (ESTs) (Fig. 1). RFLP probes refer to DNA sequence tags used to distinguish size differences or presence/absence polymorphism among the restricted fragments, while ESTs refer to cDNA sequence of functional genes.

### SNPs from RFLP probes

RFLP probes linked to traits of interest were the initial sources for SNP discovery. For instance, amplification, sequencing and analysis of RFLP probe, Xabc465-related sequences led to the identification of a SNP-based cleaved amplified polymorphic sequence (CAPS) marker (PS10R/L2) for *Lr47,* derived from *T. speltoides* (Helguera et al. 2000). Similarly, a SNP-based CAPS marker was also made available from probe cMWG682 for detecting *Ae ventricosa* segment carrying *Lr37*/*Sr38*/*Yr17* gene cluster (Helguera et al. 2003). Likewise, the MWG798 probe contributed SNPs for marker-assisted selection (MAS) of *Sr61* stem rust resistance derived from *Thinopyrum ponticum* (Mago et al. 2019). While RFLP probes played a significant role in developing SNP-based markers for high-valued rust resistance genes derived from wild relatives of wheat, it was a tedious process in the pre-genome sequencing era due to the necessity to screen probes through DNA restriction and hybridization prior to sequencing, SNP detection and marker design.

### SNPs from expressed sequence tags (ESTs)

ESTs are short cDNA fragments of a functional gene and are approximately 300-1000 bp long. ESTs are exploited for discovering SNPs using in vitro and in silico-based approaches. The first approach involves SNP mining through cDNA synthesis and sequencing, while the other approach involves in silico mining of SNPs directly from publicly available EST and cDNA databases (Allen et al. 2011). CAPS markers derived from SNPs of wheat ESTs played a vital role in mapping ASR stem rust resistance gene *Sr35* (Zhang et al. 2010) and the widely used pleiotropic APR gene *Lr34* (Lagudah et al. 2006). Although ESTs have been an essential repository for identifying SNPs, being from the genic regions, they represent a very small fraction of the genome.

### SNPs from genomic libraries

Genomic libraries refer to DNA collection where short to long DNA fragments representing the genome or cDNA of an organism are cloned into a DNA carrier [plasmids or on bacterial artificial chromosomes (BAC)] and maintained in bacterial cultures. Several species-specific BAC libraries for *T. monococcum*, *T. dicoccoides*, *T. urartu*, *T. aestivum*, *Ae. taushii* and *Ae. speltoides* are available, which serve as valuable resources in wheat genomics (Nilmalgoda et al. 2003; Janda et al. 2004, 2006; Ling and Chen 2005; Gupta et al. 2008). Prior to whole genome sequencing (WGS), these genomic libraries were the primary source for developing physical maps and cloning disease resistance genes. Subsequently, genomic clones were also critical for identifying SNP markers linked to rust resistance. Screening and sequencing clones from genomic libraries was slow and tedious, particularly in wheat with highly repetitive DNA and polyploid genomes. But, with the successful isolation of individual chromosomes, the complexity was reduced to chromosome level by generating chromosome-specific libraries (Safár et al. 2004). Additionally, repeated screening of the libraries to detect specific clones was reduced through multidimensional pooling of the clones. A good example is the identification of 195,631 D genome-specific SNPs from the sequencing of 461,706 BAC clones of *Ae. tauschii* accession AL8/78, the D genome progenitor of bread wheat. Further, these SNP markers were genetically mapped using a mapping population derived from crossing *Ae. tauschii* accessions AL8/78 and AS75 and used in mapping of the stem rust resistance gene *SrTA10187* (Luo et al. 2013; Wiersma et al. 2016). Subsequently, the generation of chromosome 3B-specific BAC libraries yielded SNPs that assisted in fine-scale mapping of widely deployed adult plant stem rust resistance gene *Sr2* on chromosome 3B (Mago et al. 2014). Although laborious, low throughput and cost-intensive, the genomic library-based approaches played a significant role in SNP identification during the pre-genome sequencing era.

### SNP discovery in post-wheat genome sequencing era

Soon after the workshop on wheat genome sequencing in Washington, USA, in November 2003, efforts were made to sequence the genome of the wheat variety Chinese Spring, which is used widely for genetic studies. Although the first comprehensive assembly of Chinese Spring was released in 2012, it was highly fragmented (Brenchley et al. 2012). However, the successful assembling of Chromosome 3B of wheat through the generation of chromosome-specific BAC libraries initiated the formation of the International Wheat Sequencing Consortium (IWGSC) that led to the release of near-complete chromosome-level reference of Chinese Spring (IWGSC 2018). Similarly, reference genome was also made available for tetraploid and diploid ancestral species of wheat through the sequencing of wild emmer (AABB) “Zavitan’ (Avni et al. 2017) and *Ae. tauschii* (DD) accession AL8/78 (Luo et al. 2017; Zhao et al. 2017). Thus, the rapid release of reference genomes paved the way for the increasing discovery of SNPs and high-throughput arrays for mapping key traits including rust resistance in wheat (Fig. 1).

### SNPs from whole genome short-read sequencing

Genomic sequences generated using short-read sequencing platforms detected millions of SNPs from genic, repetitive and non-repetitive intergenic genome regions. For instance, the sequencing of two *Ae. tauschii* accessions AL8/78 and AS75 with Roche454 and SOLID captured 195,631, 155,580 and 145,907 SNPs in gene sequences, uncharacterized non-repetitive regions and repeat junctions of D genome, respectively (You et al. 2011). Similarly, the generation of a high-quality reference sequence of *T. urartu* (accession G1812), the A genome diploid wheat using BAC library, WGS and optical mapping resulted in the identification of 541,849 A genome-specific SNPs (Ling et al. 2018). In parallel, the WGS of eight elite wheat lines identified 3.3 million SNPs where 41, 49 and 10% were located on A, B and D genomes, respectively (Rimbert et al. 2018). WGS is also helpful in detecting SNPs specific to alien segments introgressed from wild species such as *Ambylopyrum* *muticum* into bread wheat (Coombes et al. 2023).

### SNPs from specific chromosome isolation and sequencing

While WGS may be a feasible technique for organisms with smaller genome sizes, it remains a costly affair for polyploid crops such as wheat with large genome sizes. Additional complexity arrives due to the high similarity between the sequences of the three homoeologous genomes. However, with the successful flow sorting of specific chromosomes, the intricacy was reduced as demonstrated through the isolation and sequencing of chromosome 3B, where 1,835,214 SNPs specific to chromosome 3B were identified from wheat cultivars Arina and Forno (Shatalina et al. 2013). SNPs from flow-sorted chromosome 4B of VL404 and WL711 have helped in increasing the marker density and resolution of the *Lr49* region (Nsabiyera et al. 2020). Similarly, sequencing of recombinant chromosome 5D/5U from wheat-*Ae. umbellulata* introgression line identified 5U and 5D chromosome-specific SNPs for mapping *Lr76* and *Yr70* resistance genes (Bansal et al. 2020).

### SNPs from genotyping-by-sequencing

SNP discovery through WGS also requires sequencing of more than one variety or species which remains expensive and tedious. Further genetic studies involving QTL mapping, association studies and diversity analysis hardly require the whole sets of SNPs. Hence, the genotyping-by-sequencing (GBS) strategy was introduced, wherein only a subset of regions from the genome is focused through complexity reduction or targeted enrichment approaches. There are more than a dozen techniques to reduce complexity (Bhatia et al. 2013; Scheben et al. 2017). Among these, the most popular one involves sequencing of DNA fragments generated using single restriction enzyme like *Ape*KI (Elshire et al. 2011; Trebbi et al. 2011). A restriction site-associated DNA sequencing (Radseq) approach was used to detect and map 430,979 SNPs between two *T. urartu* accessions G1812 and G3146 (Ling et al. 2018). Subsequently, a two-enzyme-based restriction approach (Poland et al. 2012) involving rare and frequent cutter enzymes was also used to map rust resistance genes like *Lr27/Sr2/Yr30, Lr37, Lr46/Yr29/Sr58* (Rauf et al. 2022)*, Lr81* (Xu et al. 2022), *Sr6* (Mourad et al. 2018) and *Sr17* (Megerssa et al. 2022). Recently, a three-enzyme strategy called 3D-GBS has been introduced and tested in soybean, where the complexity is reduced to four-fold compared to the single enzyme *Ape*KI strategy (de Ronne et al. 2023) and could be extended to wheat.

### SNPs from transcriptomes

Transcriptome refers to RNA molecules (such as messenger, non-coding and small RNAs) that represent a small proportion of large genomes, such as in wheat. Due to its reduced size, transcriptome datasets have also been exploited for mining SNPs. Initially, hybridization and sequencing-based approaches were used to quantify and map transcripts. For example, using transcriptome data and serial analysis of gene expression (SAGE) technique, SNPs linked with *Lr28* resistance were detected from wheat cultivar HD2329 (Chandra et al. 2017). However, this approach was expensive and produced short tags that could not be mapped onto the reference genome. RNA sequencing (RNAseq) overcomes this limitation by allowing both quantification and mapping of transcriptomes (Wang et al. 2009). SNPs were detected with an average density of one per 569 bp from transcriptome reads of three wheat cultivars viz*.,* Excalibur, RAC875 and Kukri (Lai et al. 2012).  The in silico mining of publicly available transcriptome data has also enhanced the SNP discovery process in wheat. Besides hexaploid wheat, the RNAseq approach has also been utilized to characterize SNPs from diploid and tetraploid relatives of wheat. RNAseq data from two *Ae. tauschii* accessions belonging to two major lineages resulted in the identification of ~ 10K non-redundant D genome-specific SNPs (Iehisa et al. 2012, 2014). A total of 144,806 high-quality SNPs were discovered from the sequencing of 22,841 expressed genes of 147 *T. urartu* accessions (Ling et al. 2018). Similarly, RNAseq reads from 18 durum accessions and an emmer wheat accession led to the identification of 52,646 SNPs (Wang et al. 2014). Recently, bulked segregant analysis was combined with RNAseq (BSR-seq), where sequencing of RNA from resistant and susceptible bulks was used to detect SNPs and candidate genes for rust resistance as demonstrated for *Yr15* (Ramirez-Gonzalez et al. 2015) and *SrTM4* (Li et al. 2023) genes.

### SNPs from exome capture

Exome refers to coding sequences (also called exons) present in a genome and are selectively captured, sequenced and analysed using probes from exons, while RNAseq predicts the coding sequences of expressed genes only. Exome capturing was first applied in tetraploid wheat species, *T. dicoccoides* and *T. durum*, targeting 3497 genes where 4386 SNPs were identified (Saintenac et al. 2011). Screening of eight UK wheat varieties using a Nimblegen array designed to capture and characterize 50% of wheat exome (84 Mb) detected 511,439 SNPs, of which 99,945 were categorized as varietal SNPs based on their ability to distinguish two or more varieties (Winfield et al. 2012). Later, the Nimblegen array-based capturing was extended to a large-scale screening of wheat exome from 43 accessions including cultivated wheat and its wild relatives, generating 921,705 SNPs (Winfield et al. 2016). The array was also used to identify SNPs linked to stripe rust resistance gene *Yr78* (Dang et al. 2022). While probes are designed to target exons, non-target variants are also observed contributing to a significantly increased number of SNPs discovered compared to RNAseq, as Esposito et al. (2022) found that only 26% of the SNPs identified were in exons, while the rest were from intergenic regions. Although RNAseq and exome capturing are economical compared to WGS and GBS, the SNPs discovered are from conserved gene clusters and are insufficient for the construction of high-resolution genetic maps, which require uniformly distributed genome-wide markers.

### SNPs from pan-genome analysis

Soon after the release of reference genomes of wheat and its wild relatives (Avni et al. 2017; Zhao et al. 2017; Zimin et al. 2017), genomes of multiple wheat lines were decoded subsequently to generate pan-genome. Montenegro et al. (2017) constructed the first pan-genome assembly for wheat using a WGS dataset of 18 cultivars, wherein a total of 36.4 million SNPs were identified in addition to other structural variations in genes/genomic regions. Subsequently, the ‘10 + wheat genome project’ generated a pan-genome assembly of 10 hexaploid wheat cultivars viz*.,* Arina*LrFor*, Jagger, Julius, LongReach Lancer, CDC Landmark, Mace, Norin61, SY Mattis, CDC Stanley, PI190962 (spelt wheat) and scaffold assemblies of five UK wheat lines, viz*.* Cadenza, Claire, Paragon, Robigus and Weebill1 (Walkowiak et al. 2020). Here, in addition to multiple paired-end Illumina sequencing, 10X Genomics Chromium and Hi-C platforms were used to generate chromosome-level pan-genome assemblies. Using the 10 + wheat pan-genome and haplotype analysis, Dang et al. (2022) detected SNPs associated with stripe rust resistance gene *Yr78*.

## SNP genotyping

Large genome size, polyploid nature, gene duplications, low sequence divergence within coding regions and mutations in probe/primer annealing sites have complicated SNP genotyping in wheat. On the contrary, abundance, highly polymorphic nature, easy transferability across different platforms and successful conversion rates ranging from 50 to 97% (Semagn et al. 2014), have made SNPs a trending marker system for wheat genotyping. Prior to the NGS era, SNP genotyping was based on a few low throughput gel-based assays such as CAPS markers. However, millions of SNPs discovered through NGS pipelines warranted the need for high-throughput genotyping assays. The SNP arrays and NGS-based genotyping assays such as GBS have become essential genotyping platforms in current wheat breeding programs, where many genome-wide markers are required for constructing high-resolution genetic maps, QTL mapping, GWAS and genomic selection. Once significant marker-trait associations are identified, uniplex genotyping assays like KASP serve as an integral part of MAS programs where tightly linked markers are specifically selected for trait introgression into elite genetic backgrounds.

### SNP arrays

In addition to the large-scale SNP discovery, the increasing use of SNP markers for wheat improvement is attributed to the rapid progress in developing high-throughput SNP genotyping arrays. Apart from its cost-effective nature, arrays can also be custom designed for targeting SNPs of specific regions or genes of a genome. For polyploids like wheat, Affymetrix and Illumina-based techniques are the two widely used platforms for developing SNP arrays. While both the systems work based on oligonucleotide probe and hybridization-based capturing of DNA fragments related to the targeted SNPs, they vary with the length of the probes: 25-mer for Affymetrix and 50-mer for Illumina (LaFramboise 2009; You et al. 2018). Using these platforms, a series of SNP arrays have been developed and are currently used for wheat improvement including resistance to rust diseases.

#### Wheat 9K Illumina iSelect SNP array

Developed in 2013, this 9K SNP array is one of the first high-density genotyping arrays developed for wheat. The array consists of 9000 SNPs pooled from three different sources viz*.,* SNPs identified from reference transcripts of nine cultivated wheat accessions, SNPs from a panel of 20 landraces and genic SNPs identified from parents of the SynOp mapping population (Cavanagh et al. 2013). This bead chip array was validated by genotyping a diverse panel of 2994 hexaploid wheat accessions and a consensus genetic map comprising 7504 loci was built using six biparental mapping populations and a MAGIC population (Cavanagh et al. 2013). The utility of this array was demonstrated through the identification of high-density inter-varietal SNPs (Lai et al. 2015), assessing population structure and genetic diversity (Würschum et al. 2013), QTL mapping, association analysis and genomic selection for yield-related traits (Hao et al. 2017; Liu et al. 2020) and disease resistance (Bajgain et al. 2015, 2016). In the case of rust resistance, the 9K array was used to map *Lr67*, *Lr74*, *Lr.ace-4A*, *Sr7a*, *Sr12*, *Sr25*, *Sr56*, *Yr5*, *Yr78* and *Yr82* rust resistance genes (Supplementary Table 1a, 1b and 1c). Further, the widely used pleiotropic and triple rust resistance gene, *Lr34*/*Sr57*/*Yr18* was also mapped using the 9K array. Additionally, the array enabled the detection of novel QTLs for all-stage stem rust resistance in 7A (Pujol et al. 2015) and for adult plant resistance in 6D (Bajgain et al. 2015) and 7A (Aoun et al. 2019) chromosomes.

#### Illumina 90K iSelect array

The iSelect90K array is a custom-designed array comprising 81,587 functional SNPs, discovered from a transcriptome study involving 19 bread wheat and 18 durum accessions (Wang et al. 2014). The array was used to map 46,977 functional SNPs by genotyping eight DH mapping populations. Further, the array was evaluated for diversity studies involving 550 hexaploid and 55 tetraploid wheat accessions including landraces and cultivars of different geographic origins. Mapping of *Lr16*, *Lr33*, *Lr 2K38*, *Lr48*, *Lr64*, *Lr74*, *Lr77*, *Lr79*, *Lr80*, *Lr82*, *Sr5*, *Sr8a*/*Sr8155B1*, *Sr883-2B*, *Sr9h*, *Sr11*, *Sr12*, *Sr13*, *Sr14*, *Sr15*, *Sr16*, *Sr22*, *Sr26*, *Sr42*, *Sr60*, *Sr63*, *SrKN*, *Yr29*, *Yr66*, *Yr67* and *Yr71* rust resistance genes was undertaken using 90K iSelect array (Supplementary Table 1a, 1b and 1c).

#### Wheat 15K SNP array

The wheat 15K SNP array comprising 13,261 SNPs was derived from a 90K array based on the genotypic data of more than 2000 genotypes, including European and worldwide wheat lines (Boeven et al. 2016; Soleimani et al. 2020). Subsequently, the array was validated using 204 winter bread wheat varieties and association mapping analysis (Boeven et al. 2016). The cost-effective 15K SNP array was used for mapping the *Lr21* locus in *Ae. tauschii* (Naz et al. 2021).

#### Axiom® HD Wheat genotyping (820K) array

SNPs from 9, 15 and 90K arrays are inadequate to predict allelic diversity present in the secondary and tertiary gene pools (Rasheed and Xia 2019). Thereby, the Axiom® HD Wheat genotyping (820K) array was introduced to overcome this limitation by incorporating 819,571 SNPs identified through exome capturing of 43 wheat accessions and wild relatives belonging to diploid, tetraploid, hexaploid and decaploid species. The array was validated by genotyping 475 accessions representing A, B and D genomes and individuals from three mapping populations. About 289,859 SNPs were physically mapped using cytogenetic stocks, and 56,505 markers were genetically mapped onto a consensus map spanning 3739 cM in length using three mapping populations. The axiom array was further validated for its utility in analysing population structure and diversity, as well as in detecting and mapping novel introgressions onto the wheat chromosomes (Winfield et al. 2016). However, this array finds limited utility in a hexaploid wheat breeding program since most markers are from wheat relatives, derived from coding regions of genes that represent a small fraction of the wheat genome and are not amenable for cost-effective genotyping (Allen et al. 2017). Axiom® HD wheat genotyping (820K) array was used for mapping leaf rust resistance gene *Lr32* (Sharma et al. 2023).

#### Wheat Breeders’ 35K Axiom array

To have a cost-effective high-throughput genotyping platform, a subset of 35,143 SNP markers from the 820K array were selected based on their even distribution in the genome and high level of polymorphism to design the Wheat Breeders’ 35K Axiom array. By genotyping five mapping populations, 62.6% of these SNPs were genetically mapped. Further, screening a unique and elite collection of 1,779 hexaploid accessions, including those from Gediflux and Watkins global landrace collections, nullisomic and monosomic cytogenetic stocks, the array demonstrated its utility in high-density genetic mapping, diversity and genomic rearrangements in hexaploid wheat (Allen et al. 2017). Genomic regions of rust resistance genes such as *LrTs276-2*, *LrM*, *SrH*, *SrY* and *Yr29*/*Lr46* as well as novel QTL (QYrcw.nwafu.3BS) were mapped using 35K Axiom array (Supplementary Table 1a, 1b and 1c).

#### Axiom®wheat 660K SNP array

The Axiom®wheat 660KSNP array was designed by the Chinese Academy of Agricultural Sciences (CAAS). From a robust collection of 51 million genome-specific SNPs generated through GBS (78 accessions), RNAseq (32 accessions) and resequencing data from different wheat genomes, four 623K arrays were designed initially and screened using 192 wheat accessions. The highly polymorphic SNPs were then used to generate the high-density Axiom®wheat 660K SNP array for commercial purposes. With a capacity to detect 100,000 genes (almost all wheat genes) and 78% of the SNPs genetically mapped (Cui et al. 2017), the 660K array has become a potential genotyping platform for diversity, haplotype analysis and investigating the genetic basis of agronomically important traits in wheat (Jin et al. 2016; Yang et al. 2019; Sun et al. 2020). All-stage resistance QTL, QYrXN3517-2BL was mapped using 660K SNP array (Huang et al. 2023). But like the Axiom® HD Wheat genotyping (820K), the use of 660K SNP array was not a cost-effective approach.

#### Wheat 55K SNP array

The wheat 55K SNP array comprising 53,063 markers derived from the 660K SNP array and associated with important agronomic traits was designed jointly by the CAAS and Affymetrix. Through 55K SNP array, the genotyping cost is cut down to one-third of the 660K SNP array, and its utility was validated for constructing high-density genetic maps and QTL mapping (Liu et al. 2018; Fan et al. 2022). The array was also useful in mapping *Yr30*/*Sr2* and novel QTLs including QLr.hebau-5AL/QYr.hebau-5AL, QLr.hebau-3BL, QYr.hebau-5AL, QYr.hebau-4BS, QYr.hebau-6BS, QYr.nwafu-7BL, QYr.gaas.2A and QYr.gaas.6A (Zhang et al. 2019b; Huang et al. 2019; Gebrewahid et al. 2020; Cheng et al. 2022).

#### 18K Axiom^TM^ 384 layout array

The 18K Axiom^TM^ 384 layout array synthesized by Affymetrix, comprises 18,101 SNPs derived from a high density 420K Axiom array developed under the Collaborative French Breed Wheat Project. The uniqueness of this array is that only reproducible and co-dominant SNPs covering the entire genome were selectively included based on the characterisation of 200 wheat accessions from diverse geographical regions. The utility of the array in generating a consensus linkage map was demonstrated by genotyping nine DH populations developed from Australian wheat germplasm (Norman et al. 2017).

#### TaBW280K chip array

The previously described arrays include SNPs discovered primarily from transcriptomes, exomes and GBS. The TaBW280K chip was designed using a small subset (280,226 SNPs) of three million SNPs identified through the WGS of wheat. It is a high throughput genotyping array comprising of 225,596 intergenic and 54,280 genic SNPs. The array has been used to construct a high-density genetic linkage map comprising of 83,721 markers covering a length of 3308 cM (Rimbert et al. 2018).

#### Wheat 50K (TriticumTraitBreed) array

Recently, Rasheed and Xia (2019) reported a customized Wheat 50K array (TriticumTraitBreed array) based on the high-quality SNPs selected from the Wheat 35, 90 and 660K SNP chips. Around 135 functional markers, and 700 SNPs tightly linked with known QTLs were included in this array.

#### 10K *Ae. tauschii* Infinium SNP array

The 10K *Ae. tauschii* Infinium SNP array is a species-specific array developed by selecting 10,000 SNPs from a pool of nearly 200,000 SNPs identified between *Ae. tauschii* accessions AL8/78 and AS75, out of which 9,485 were from functional assays. A small fraction of SNPs (515 SNPs) was in wheat ESTs and have been assigned to linkage groups on the AL8/78 × AS75 genetic map (Luo et al. 2013). Rust resistance genes *Lr42* and *YrAs2388* of *Ae. tauschii* were characterized using this D genome-specific arrays (Gill et al. 2019).

#### Illumina Infinium wheat barley 40K SNP array

The recently developed wheat barley 40K SNP array is a multi-species array that was designed to enable robust imputation SNP genotyping with high accuracy. It comprises 14,261 and 25,363 SNP markers, respectively, from barley and wheat that are linked to key agronomic traits. The SNP markers for wheat were based on exome sequencing of 1041 bread wheat accessions, while the SNPs for barley were selected based on exome sequencing of 267 accessions and whole genome sequencing of 117 accessions from the Intergrain commercial barley breeding programme (Keeble-Gagnère et al. 2021). Since it permits the hybridization of multiple samples on a single array, the wheat barley 40K SNP array serves as a common and cost-effective genotyping platform for both crop species, finding broad applications in genome-wide association studies and genomic selection.

Given the wide range of arrays, breeders must make a careful choice depending on the germplasm panel used and the nature of the genetic analysis required. Moreover, the array-based SNP markers introduce an ascertainment bias that can underestimate diversity and genome prediction abilities (Chu et al. 2020).

#### *Triticum aestivum* next generation (TaNG) array

The recently developed TaNG array consists of 43,372 SNP markers, sourced from the whole genome sequence of 204 elite wheat lines and 111 Watkins wheat landraces. These SNPs were selected based on the ability to distinguish varieties and conversion into definitive markers (Burridge et al. 2024).

### Next-generation sequencing (NGS)-based SNP genotyping systems

Among NGS-based genotyping systems, GBS involving one-enzyme (Elshire et al. 2011) and two-enzyme strategies (Poland et al. 2012) has been rewarding, particularly in identifying novel genetic loci associated with rust resistance (Supplementary Table 2 and 3). Through exhaustive QTL mapping and genome-wide association studies, it paved the way for the rapid selection of rust resistance through MAS. Targeted GBS (tGBS), intended for targeting and saturating specific genomic regions, is an extended innovation of GBS that is effective in characterising several thousand markers across a large number of samples. The tGBS approach has been employed for mapping all-stage stripe rust resistance genes *YrAw12* (Baranwal et al. 2021), *YrPAK* (Tariq et al. 2021) and an adult plant stripe rust resistance gene *Yr75* (Kanwal et al. 2021).

 Compared to SNP arrays, GBS introduces less ascertainment bias and is more reliable in the prediction of rare alleles that enable the unravelling of molecular diversity in the gene pool (Elshire et al. 2011; Rasheed et al. 2017; Chu et al. 2020). Further, it does not require prior sequence information or targeted probe sets. However, it involves a complex two-step library preparation comprising restriction enzyme digestion and adapter ligation. This limitation has been overcome by simplified NGS library preparation protocols, such as Nextera, that enable simultaneous DNA fragmentation and adaptor ligation in a single step using a transposon complex (Caruccio 2011). Whole genome coverage to varying levels can be achieved by low-depth (1-2x) sequencing of these libraries, referred to as Skim sequencing (Skim-Seq). The utility of Skim-Seq approach as a genotyping platform and as a tool for genomics-assisted breeding has been demonstrated using DH populations and cytogenetic stocks in wheat (Adhikari et al. 2022).

Despite being robust, a significant proportion of SNPs (18–33%) detected in these multiplex platforms are discarded owing to factors such as missing data, minor allele frequency, weak amplification, ambiguity in heterozygote calling and lack of polymorphism in the panel surveyed (Rasheed et al. 2017). In addition, the high cost per sample and the substantial time involved limit the application of these NGS-based SNP genotyping platforms, specifically in areas where fewer samples need to be screened with relatively low to moderate marker density. Under such situations, uniplex SNP genotyping platforms are considered to be efficient and economical.

### Uniplex SNP genotyping platforms

#### Cleaved amplified polymorphic sequences (CAPS)

Sequence polymorphisms such as SNPs that introduce changes in the recognition site of restriction enzymes are useful for developing CAPS markers. DNA sequence spanning these polymorphic restriction sites is PCR amplified using sequence-specific primers and subjected to restriction digestion to detect the polymorphism based on cleavage. DNA fragments captured through RFLP or AFLP analysis and linked with disease resistance are valuable sources for developing CAPS markers. ASR leaf rust resistance gene *Lr51* introgressed from *T. speltoides* and a pleiotropic APR gene *Lr37 (Sr38/Yr17*) introgressed from *Ae. ventricosa* into chromosomes 1B and 2A of bread wheat, respectively, was mapped using CAPS markers (Helguera et al. 2003, 2005). Based on haplotype analysis in diverse germplasm, a diagnostic CAPS marker (GLP-1/2 CAPs) developed from a SNP lying in the promoter region of the *TaGLP* gene was used for genotyping the stem rust resistance locus *Sr2* (Mago et al. 2014). Subsequently, the closely linked csSR2 marker developed for *Sr2* was also based on CAPS assay (Mago et al. 2011).

#### Taqman assay

Taqman assay, also known as 5’ nuclease assay, is a real-time PCR-based assay for SNP genotyping. It comprises two differentially labelled probes and an unlabelled primer pair to amplify the target region. When the probes are intact, the fluorescence emitted by the reporter dye is suppressed by the quencher dye. Specific annealing of the probe complementary to the target sequence, followed by cleaving of the hybridized probe by exonuclease activity of Taq polymerase, results in fluorescence, indicating the specific allele amplified. The Taqman assay has been used for MAS of the adult plant leaf rust resistance gene *Lr2K38* located on chromosome 1A (Sapkota et al. 2020).

#### Kompetitive allele-specific PCR (KASP) assay

While GBS and array platforms are robust and advantageous in mapping genes and QTLs, screening a small subset of trait-linked SNP markers using these platforms is an expensive exercise. Once identified from GBS and chip assays, SNPs linked with key agronomic traits are converted into KASP assay for marker-assisted breeding programs. The KASP assay is an allele-specific assay that uses a universal fluorescence resonant energy transfer (FRET) cassette to enable bi-allelic scoring of SNPs and InDels at a specific locus. Several such KASP markers designed from informative SNPs are used as diagnostic markers in MAS for numerous major genes and QTLs associated with resistance to leaf rust, stem rust and stripe rust (Supplementary Table 1a, 1b, 1c and 2). Due to its speed, simplicity and uniplex nature of detecting SNPs, the KASP assay is rapidly replacing SSR and other gel-based marker systems. However, a common problem encountered while converting array-based SNP to KASP markers in wheat is the frequent false calling of heterozygous genotypes and lack of locus specificity. This results from SNPs within the polyploid genomes exhibiting inter-homologue polymorphism in some individuals which makes it difficult to distinguish homozygotes from heterozygotes. Hence, to overcome this pitfall and ensure the successful conversion of SNP into a KASP marker, Makhoul et al. (2020) insisted on the alignment of SNP probes with the reference genome, sanger sequencing and visual KASP primer placement as critical factors for consideration.

#### Semi-thermal asymmetric reverse PCR (STARP)

STARP is another novel method of genotyping individual SNPs (Long et al. 2017). Basically, STARP includes competitive amplification of two SNP alleles using two universal priming element adjustable primers and one group of three locus-specific primers: two asymmetrically modified allele-specific primers and their common reverse primer. The resulting PCR products can be visualized either by gel-based or florescence-based methods for detecting SNP alleles. This method overcomes the limitations and combines major advantages of the current SNP genotyping technologies in terms of accuracy, flexibility, simplicity and cost-effectiveness. For instance, the traditional allele-specific PCR (Myakishev et al. 2001) shows a low SNP detection rate and the KASP, which is an improved allele-specific PCR, involves higher operational cost due to dependency on the developer (Middlesex, UK; http://www.lgcgroup.com) for PCR reagents and allele-specific primers, and the need for sophisticated equipment such as real-time PCR machines or fluorescence readers. In contrast, STARP can be performed using standard PCR conditions and adopted in both conventional PAGE and high-throughput genotyping platforms; therefore, it can be followed across laboratories with minimum resources. STARP markers have been used for MAS in wheat rust resistance breeding programmes. Sharma et al. (2019) designed two SNP-based dominant STARP markers for the stem rust resistance gene *Sr883*-*2B* and a co-dominant STARP marker for *Sr883-6A*, a likely allele of *Sr13* gene derived from the cultivated emmer accession PI193883. These markers were validated for their utility for MAS using a panel of 48 durum and cultivated wheat cultivars. Among the three, the co-dominant STARP marker (rwgsnp7) was found to be effective for a gel-free assay system.

## Creating artificial SNPs for rust resistance analysis

### Mutation-based strategies

Mutation breeding has become a valuable method to analyse genes that are linked to agronomically important traits such as rust resistance. Targeting induced local lesions in genomes (TILLING) has been deployed as a reverse genetic tool to identify genotypes carrying mutations on genes linked to key traits (McCallum et al. 2000). TILLING populations carrying artificial mutations add value to the existing germplasm resources in terms of creating new markers and are developed for both tetraploid and hexaploid wheat (Chen et al. 2012, 2014; Rawat et al. 2012, 2019; Colasuonno et al. 2016; Mo et al. 2018; Richaud et al. 2018; Harrington et al. 2019; Madsen and Brinch-Pedersen 2020). Particularly, the Kronos and Cadenza TILLING populations have been extensively used by wheat breeders for functional characterization of agronomically important genes (Chen et al. 2012; Simmonds et al. 2016; Krasileva et al. 2017; Uauy 2017; Mo et al. 2018; Richaud et al. 2018; Marchal et al. 2018; Harrington et al. 2019; Chia et al. 2020; Ajaz et al. 2021; Debernardi et al. 2022; Desjardins et al. 2022). The Kronos TILLING population comprising of 1,536 mutant lines was generated using ethyl methane sulfonate (EMS) treatment of a tetraploid durum variety Kronos (Uauy et al. 2009). The Cadenza TILLING population comprising of 3,750 mutant lines was also developed through EMS mutagenesis of ‘Cadenza’, a hexaploid Chinese Spring cultivar (Rakszegi et al. 2010). In addition to these TILLING resources, A genome and D genome-specific TILLING populations have also been developed from *T. monococcum* and *Ae. tauschii*, respectively (Rawat et al. 2012, 2018).

Although initially developed as a reverse genetic tool, TILLING also finds application in forward genetics to characterize the novel SNPs artificially induced through mutation. The cost-cutting NGS techniques have opened avenues to scan the genome of interesting mutant lines from the TILLING populations. The entire set of Kronos TILLING population was exome sequenced, and a mutant line T4-3822 harbouring 1,874 EMS-induced SNPs was identified (Krasileva et al. 2017). The NGS-based exome capture assay of 11 mutant lines from a TILLING population of NN-Gandium-1 detected 104,779 SNPs distributed across A, B and D genomes (Hussain et al. 2018). Exome capturing and sequencing of a 2Mb region from three mutant lines of the Cadenza TILLING population detected at least 464 SNPs indicating the presence of 35 SNPs per Mb (King et al. 2015). Sequencing EMS mutants derived from the hexaploid cultivar ‘Indian’ using GBS detected 14,130 induced mutations including SNPs and InDels (Sidhu et al. 2015). Prior to such large genome survey, TILLING populations were initially limited to discovering SNPs in candidate genes. A set of 275 novel alleles were detected for 11 target genes using the mutant libraries of Kronos and a hard red spring wheat breeding line 'UC1041 + *Gpc-B1*/*Yr36*' carrying high protein content gene and partial stripe rust resistance gene (Uauy et al. 2009). Many novel allelic variants for key genes involved in starch biosynthesis, kernel hardness, carotenoid biosynthesis, head blight resistance and glyphosate tolerance have also been discovered from TILLING populations (Dong et al. 2009; Slade et al. 2012; Colasuonno et al. 2016; Li et al. 2017; Gadaleta et al. 2019; Moehs et al. 2021).

Similarly, sequencing stem rust susceptible mutant lines from *Sr35* resistant accession G2919 identified G-A mutations in the disease resistance gene *CNL9* present within the *Sr35* region. The mutation resulted in a premature stop codon producing a truncated protein (Saintenac et al. 2013). TILLING population from a wheat variety NN-Gandium-1 investigated for functional analysis of genes associated with leaf and stripe rust resistance identified a candidate SNP in *Lr21* gene on chromosome 1B. Through prediction analysis, the synonymous SNP in the nucleotide binding site (NBS) domain was found to alter the protein structure by alanine to glutamic acid substitution (Hussain et al. 2018). A branched-chain amino acid transferase in wheat (TaBCAT1) is known to be involved in a salicylic acid-dependent defence activation pathway. By analysing loss of function mutants from the Kronos TILLING population, two TaBCAT1 disruption mutant lines were identified. One mutant from A genome (Kronos2898) encoding a stop codon and the other from B genome (Kronos860) encoding for a truncated protein showed reduced susceptibility to *Pst* isolates causing leaf rust, thereby establishing the role of TaBCAT1 in positively regulating wheat rust susceptibility (Corredor-Moreno et al. 2021). The mounting mutant resources for TILLING coupled with NGS techniques remain less exploited for functional characterization of several rust resistance genes identified in wheat. A major limitation in using these TILLING populations is attributed to the random mutations, which demand extensive screening to identify ‘loss or gain of function’ mutants making it a laborious and time-consuming exercise. These limitations can be overcome through site-directed mutagenesis using gene editing techniques.

### Gene expression modifications (SNPs in promoters)

Promoter regions lying upstream of the genes contain specific motifs that act as *cis*-regulatory elements that are required for the binding of transcription factors to initiate the transcription process. SNPs in these *cis*-regulatory elements of promoters can alter the nature and rate of binding of these transcription factors, thereby affecting gene expression. Several novel SNPs have been identified in the promoters of yield-related genes in wheat viz., *TaGw2-6a*, *TaCWI-4* and *TaCYP785* (Jaiswal et al. 2015; Jiang et al. 2015; Guo et al. 2022) that were responsible for differential gene regulation. The *Sr2* locus has been physically mapped on chromosome 3B of wheat cultivar Hope. Interestingly, this locus did not belong to the NLR family but rather contained Germin-like proteins (GLPs) encoding candidate genes associated with disease resistance. Based on a haplotype analysis between the *Sr2*-containing Hope cultivar and the non-*Sr2* wheat cultivar Chinese Spring, several SNPs and InDels were detected only in the promoter region and not in the coding regions of the genes, which speculates the role of these SNPs in disease resistance expression (Mago et al. 2014).

## Outlook

The post-genomics era has witnessed gold-standard technologies that are accelerating the wheat breeding at an unprecedented rate.

### Haplotype block-based approaches for resistance detection and stacking

Phenotype being a complex expression of the interaction of genes and environment, the concept of trait-based selection is now being extended from favourable alleles to haplotypes in disease resistance breeding. For instance, a GWAS analysis showed that selection of the TraesCS2B01G513 haplotype containing four natural polymorphisms in a gene that encodes for serine/threonine protein kinase (STPK) can effectively improve resistance to yellow rust in wheat cultivars (Wu et al. 2021). While the traditional GWAS identifies individual SNPs as causal variants associated with the trait, adopting haplotype block analysis where the specific pattern of a group of SNPs associated with traits such as rust resistance was predicted and utilized in crop breeding. This requires the integration of sophisticated machine learning algorithms and predictive models to bring about dimensionality reduction of data sets and augment the detecting power of novel rust resistance loci through GWAS (Difabachew et al. 2023). Machine learning involving either a reference-based imputation (such as BEAGLE, IMPUTE 5, TOPmed) or a reference-free imputation (such as the Random Forest and neural networks) can improve the efficiency of genotype calling, thereby increasing the statistical power of association analysis to identify significant marker-trait associations (Song et al. 2020).

### Pan-genome assembly for haplotype discovery

Parallelly, third-generation sequencing techniques, such as single molecule real-time (SMRT) sequencing and nanopore sequencing that can generate long reads, make genome assembly and reconstruction easier for pan-genome analysis. However, the longer read length comes at the compromise of accuracy and demands error correction and DNA polishing. Recently, Hifi sequencing has become a gold standard that meets the dual demand of long read length and accuracy (Hon et al. 2020). Pan-genome assembly, haplotype phasing and variant calling are therefore no longer a daunting task. Further, with improvements in deep learning and machine learning models, maximizing genetic gains through genomic selection based on chromosome stacking approaches that involve the selection of superior parents carrying chromosomal segments harbouring desirable haplotype blocks in wheat hybridization programmes are also gaining momentum (Villiers et al. 2024). Further reduced cost for short DNA read sequencing enables sequencing of large germplasm sets such as Watkins wheat landrace collection, thereby detecting haplotype blocks associated with key traits such as rust resistance (Cheng et al. 2024).

### Integrating machine learning and digital imaging to accelerate resistance phenotyping

With developments in high-throughput phenomics based on image acquisition through unmanned air vehicles and genotyping based on binary SNP encoding, the futuristic approach aims to integrate these data using machine learning techniques to train model sets for predicting the phenotypes from genotypes (Fig. 1). However, phenotypic plasticity of plants in varying environmental conditions and pathogen interactions poses challenges that need to be addressed while deploying ‘genotype to phenotype’ models.

Yet, precision and accuracy in dissecting the complex phenotypes and strengthening the phenomics platform for rust resistance screening at the same pace as genomics for SNP discovery and genotyping would speed up rust resistance breeding in wheat to ensure a secured food supply to meet the rising human needs.

## Supplementary Information

Below is the link to the electronic supplementary material.

Supplementary file1 (DOCX 32 KB)

Supplementary file2 (DOCX 32 KB)

Supplementary file3 (DOCX 30 KB)

Supplementary file4 (DOCX 36 KB)

Supplementary file5 (DOCX 38 KB)

## Data Availability

No datasets generated or analysed during this study.
