# Supplementary material for: Wheat improvement through advances in single nucleotide polymorphism (SNP) detection and genotyping with a special emphasis on rust resistance
Source: Theor Appl Genet. 2024 Sep 16;137(10):224. doi: 10.1007/s00122-024-04730-w (PMC11405505; doi:10.1007/s00122-024-04730-w)
Supplement: Supplementary file 1 — Supplementary file1 (DOCX 32 KB) [file 122_2024_4730_MOESM1_ESM.docx]

**Supplementary table 1a: Role of SNPs in mapping and selection of known leaf rust resistance genes**

| Genes | Type | Chromosome | SNPs (for selection) | Assay type | Flanking/  linked SNPs | Arrays/marker source | Mapping population | References |
| --- | --- | --- | --- | --- | --- | --- | --- | --- |
| *Lr13* | ASR | 2B | Lseq302 | KASP | CAUT163 - Lseq22 | SNPs/Indels through  Re-sequencing  KASP | 3908 F_2_ plants from Han 87-1 (87-1) / 7D49 and Liaochun10 /RL4031 cross | Qiu et al. (2020) |
|  | ASR | 2B | *Lr13*haplo1  *Lr13*haplo2 | KASP | WGGB618 WGGB476 | MutRenSeq | 266 RILs of cross M114 / W301 cross | Hewitt et al. (2021); Yan et al. (2021) |
| *Lr14A* | ASR | 7B | MCK161_Lr14aSec1F  MCK181_Lr14a_Sec5R |  | - | MutchromSeq | 158 RILs of Arina / Forno  2 NILs-Arina*Lr*For, Arina*Lr14a* | Kolodziej et al. (2021) |
| *Lr16* | ASR | 2B | 2BS-5194460_kwm747  2BS-5192454_kwm677 2BS-5175914_kwm847 2BS-175914_kwm849 | KASP | BS00099465_  kwm179  2BS-5157588_ kwm651 | 90 K SNP array  SNP database at CerealsDB,  Whole exome capture | 384 RILs of BW278 / AC Foremost  94 RILs of Kenyon/86ISMN 2137 cross  400 DH lines of AC Majestic / Glenlea cross  172 DH lines of RL4452 /AC Domain cross | Kassa et al. (2017) |
| *Lr21* | ASR | 1D | Lr21_GQ504819_1346_C/T | KASP | RAC875_c10925_1887 (SNP-*Lr21*)  SSLP-*Lr21* | 15K SNP array Resequencing of *Lr21* locus | 250 BC_2_F_3:6_ lines of Sy022L / Batis  140 *Ae. tauschii* accessions, North American spring wheat cultivars (67) and winter cultivars (10) | Naz et al. (2021); Neelam et al. (2013) |
| *Lr22A* | APR | 2D | SWSNP5  SWInDel4  SWInDel7 | - | SWSNP4- SWSNP6 | *de novo* sequence assembly of 2D chromosomes of CH Campala and CH Campala *Lr22a*. | 1,656 F_2_ plants derived from CH Campala *Lr22a* /CH Campala cross | Thind et al. (2017) |
| *Lr27/*  *Sr2/*  *Yr30/ QLr.umn-3B/* | APR | 3B | IWB57990  IWB6491 | KASP | chr3B_6396363  chr3B_6071517 | GBS | 176 F_4_-derived F_6_ RILs from Apav/ Copio cross | Rauf et al. (2022) |
|  |  |  |  |  | IWB57990  IWB6491 | 90K SNP array  KASP | 148 F_2_ plants Zhengmai 9023 / P10057  150 RILs of MX169 / P10057  161 F_2_ lines from AvS / P10057 | Wu et al. (2017) |
| *Lr32* | ASR | 3D | kwh147  kwh722 | KASP | kwh142  kwh355 | 90K SNP array  35K SNP array  820K SNP array | 244 DH lines and 2000 F_2_lines derived from Thatcher /BW196R cross | Sharma et al. (2023) |
| *Lr33* | ASR | 1B | kwh333  kwh335 | KASP | SNP BS00022625_51 BobWhite_c39153_131 | 90 K SNP array | 116 DH lines of KU168-2 / RL6071 cross | Che et al. (2019) |
| *Lr34/ Sr57/*  *Yr18/*  *QLr.hwwg-7DS* | APR | 7D | cSLV34  cssfr6  wMAS000003 | STS  CAPS  KASP | Lr34exon11-KASP | 9K SNP array  KASP | 127 RILs from Ning7840 / Clark cross | Lagudah et al. (2006); Lagudah et al. (2009); Li et al. (2017) |
| *Lr37/ Lr17a/ QLr.umn-2A* | ASR | 2N/2A | - | - | Traes_2AS_3381A39C6 -SNP30671452 | 90 K SNP array | 141 RILs of Jagger / ‘2174’ cross | Xue et al. (2018) |
|  |  |  |  |  | chr2A_19914469  chr2A_21005775 | GBS | 176 F_4_-derived F_6_ RILs of Apav/ Copio cross | Rauf et al. (2022) |
| *Lr2K38/ QLr.ags-1AL* | ASR | 1A | IWB20487 | Taqman | IWB20487 and  IWA4022 | 90 K SNP array | 225 RILs of AGS2038 / UGA111729 cross | Sapkota et al. (2020) |
| *Lr42* | ASR | 1D | SNP113325 and TC387992 | KASP | TC387992  WMC432 | *Ae. tauschii* 10K SNP array  KASP | 100 F_2:3_ lines each from TA2450/ TA2433 and TA2450/ TA10132 (AL8/78) crosses  234 RILs of KS93U50 / Morocco cross | Gill et al. (2019) |
|  |  |  | TaRPM1-KASP1 and TaRPM1- KASP2 | KASP | TC387992  WMC432 | RNA-seq  KASP | 140 RILs of KS93U50 / Morocco cross  56 NILs from KS93U50 / OK92G205 cross | Liu et al. (2021) |
|  |  |  | pC43 | KASP | pC43 — pC50 | BSR-Seq | 100 F_2:3_ lines of TA2433 / TA2450 cross  101 F_2:3_ lines ofTA10132 / TA2450 cross | Lin et al. (2022) |
| *Lr 46/ Yr29/ Sr58/ QLr.umn-1B/ Qlr.crc-1BL / QYr.crc-1BL* | APR | 1B | BA00138075  BA00606902 | KASP | AX-94673495  AX-95152083 | 15K SNP array  35K SNP array  50K DArT array (DArTSNPs) | 148 RILs from each of Atred#2 / Heller#1 and Atred#2/ Dunkler crosses | Megerssa et al. (2020) |
|  |  |  | - | - | chr1B_670207768 | GBS | 176 RILs from Apav/ Copio cross. | Rauf et al. (2022) |
|  |  |  | kwh404  kwh405 | KASP | Excalibur_c35888_208 Bobwhite_rep_c62955_567 | 90K SNP array  KASP | 168 DH lines from Toropi-6.4 / Thatcher cross | Rosa et al. (2019) |
| *Lr47* | ASR | 7A | KASP-Lr47-1 (S7A_93259643)  KASP-Lr47-2  (S7A_93259654) | KASP | S7A_33054479 -S7A_693104117 | GBS | 158 RILs of CI 17884 / Bainong 418 cross | Xu et al. (2021) |
| *Lr48* | ASR | 2B | IWB70147 | KASP | IWB36920 IWB72894 IWB31002 IWB39832 IWB34324 IWB70147 | 90K SNP array | 90 RILs derived from CSP44 / WL711 cross | Nsabiyera et al. (2016) |
| *Lr57/*  *Yr40*  *(M genome)Lr76/*  *Yr70*  *(U genome)* | ASR | 5D | Lr57/Yr40MAS-CAPS16 | CAPS | Lr57/Yr40MAS-CAPS16 | CAPS | 234 BC_2_F_7_ RILs of IL 393-4 / T756 cross | Bansal et al. (2020); Kuraparthy et al. (2009) |
| *Lr64* | ASR | 6A | K-IWB59855 | KASP | K-IWB59855  K-IWB72197 | 90K SNP array | 104 RILs of Thatcher / RL6149 | Kolmer et al. (2019) |
| *Lr65* | ASR | 2A | 1500-1 | Indel PCR | AltID-11 | Whole genome resequencing of Altgold | 622 F_2_ populations derived from CS / Altgold and Xuezao / Altgold | Zhang et al. (2021) |
| *Lr67* | APR | 4D | csSNP856 | KASP | csSNP275  csSNP856  csSNP754  csSNP856 | 9K SNP array  RAD tag Sequencing | 124 F_3_/F_4_ lines derived from Thatcher / RL6077 cross  148 RILs derived from Avocet / RL6077 cross | Forrest et al. (2014) |
| *Lr74/*  *QLr.hwwg-3BS.1* | APR | 3B | - | - | IWA4654   IWA1702 | 9K SNP array  KASP | 127 RILs from Ning7840 / Clark cross | Li et al. (2017) |
|  |  |  | K_IWB44132  IWB5790  IWB8467 | KASP | cs*Sr2* IWB5790 | 90K SNP array  CAPS  KASP | 100 RILs of Tc∗3/Caldwell 24-1 cross | Kolmer et al. (2018b) |
| *Lr77* | APR | 3B | - | - | IWB32805  IWB73555  IWB10344 | 90K SNP array | 113 RILs of Thatcher / SantaFe cross | Kolmer et al. (2018c) |
| *Lr78* | APR | 5D | IWA6289 | KASP | IWA6289 | 9K SNP array  90K array  KASP | 100 RILs each of Thatcher x 3/Toropi 3A12A and Thatcher x 3/Toropi 4A21A crosses | Kolmer et al. (2018a) |
| *Lr79* | ASR | 3B | KASP_31457 | KASP | 1251355  1139428 | 90 K SNP array  DArTSeq | 171 RILs of Aus26582 / Bansi | Qureshi et al. (2018) |
| *Lr80/ LrH2* | ASR | 2D | KASP_17425  KASP_17148 | KASP | KASP_17425  KASP_17148 | 90 K SNP array | 143 F_3_ lines from Agra Local/Hango-2 cross | Kumar et al. (2021) |
| *Lr81* | ASR | 2A | Xstars_KASP320 Xstars_KASP323 | KASP | Xstars_KASP320 Xstars_KASP323 | GBS | 200 F_2:3_ families of PI 470121 / Stardust cross | Xu et al. (2022) |
| *Lr82* | ASR | 2B | KASP_22131 | KASP | KASP_22131  KASP_11333 | 90K SNP array  KASP | 200 RILs of AvocetS / Aus27352 | Bariana et al. (2022) |
| *Lr83 (LrX)* | ASR | 1D | - | - | K-IWB38437-  1D_9037138 | GBS  KASP | 216 RILs from Thatcher line 78–1 /Thatcher (NIL_S_) cross | Kolmer et al. (2023); Kolmer et al. (2019) |
| *Lr.ace-4A* | ASR | 4A | - | -- | IWA232  WA1793 | 9K SNP array | 180 RILs of Rusty / PI 192051-1 cross | Aoun et al. (2019) |
| *LrTs_276-2_* | ASR | 1D | - |  | AX-94393474 | 35K SNP array | 284 F_2:3_ and BC_1_F_1_ populations derived from TSD276-2 / Agra Local cross | Dinkar et al. (2020) |
| *LrM* | ASR | 2A | - | - | SNP_AX-948171722AS, SNP_AX-945380402AS SNP_AX-945219402AS | 35 K SNP array | 318 F_2_ and F_2:3_ populations of ER9-700 / Agra Local cross | Rani et al. (2020) |
| *LrKP* | ASR | 2B | - | - | *Lrkp*2B114  *Lrkp*F299R300 | BSRseq | 114 F_3:4_ lines from KP / ZZ5389 cross | Bai et al. (2022) |

**References**

Aoun M, Kolmer JA, Rouse MN, Elias EM, Breiland M, Bulbula WD, Chao S, Acevedo M (2019) Mapping of novel leaf rust and stem rust resistance genes in the Portuguese durum wheat landrace PI 192051. Genes Genom Genet 9:2535-2547

Bai S, Pang S, Li H, Yang J, Yu H, Chen S, Wang X (2022) Broad-Spectrum Resistance to Leaf Rust in the Argentinean Wheat Cultivar “Klein Proteo” Is Controlled by *LrKP* Located on Chromosome 2BS. Agriculture 12:1836

Bansal M, Adamski NM, Toor PI, Kaur S, Molnár I, Holušová K, Vrána J, Doležel J, Valárik M, Uauy C, Chhuneja P (2020) *Aegilops umbellulata* introgression carrying leaf rust and stripe rust resistance genes *Lr76* and *Yr70* located to 9.47-Mb region on 5DS telomeric end through a combination of chromosome sorting and sequencing. Theor Appl Genet 133:903-915

Bariana HS, Babu P, Forrest KL, Park RF, Bansal UK (2022) Discovery of the new leaf rust resistance gene *Lr82* in wheat: Molecular mapping and marker development. Genes 13:964

Che M, Hiebert CW, McCartney CA, Zhang Z, McCallum BD (2019) Mapping and DNA marker development for *Lr33* from the leaf rust resistant line KU168-2. Euphytica 215:1-14

Dinkar V, Jha S, Mallick N, Niranjana M, Agarwal P, Sharma J, Vinod (2020) Molecular mapping of a new recessive wheat leaf rust resistance gene originating from *Triticum spelta*. Sci Rep 10:22113

Forrest K, Pujol V, Bulli P, Pumphrey M, Wellings C, Herrera-Foessel S, Huerta-Espino J, Singh R, Lagudah E, Hayden M, Spielmeyer W (2014) Development of a SNP marker assay for the *Lr67* gene of wheat using a genotyping by sequencing approach. Mol Breed 34:2109-2118

Gill HS, Li C, Sidhu JS, Liu W, Wilson D, Bai G, Gill BS, Sehgal SK (2019) Fine mapping of the wheat leaf rust resistance gene *Lr42*. Int J Mol Sci 20:2445

Hewitt T, Zhang J, Huang L, Upadhyaya N, Li J, Park R, Hoxha S, McIntosh R, Lagudah E, Zhang P (2021) Wheat leaf rust resistance gene *Lr13* is a specific *Ne2* allele for hybrid necrosis. Mol Plant 14:1025-1028

Kassa MT, You FM, Hiebert CW, Pozniak CJ, Fobert PR, Sharpe AG, Menzies JG, Humphreys DG, Rezac Harrison N, Fellers JP, McCallum BD, McCartney CA (2017) Highly predictive SNP markers for efficient selection of the wheat leaf rust resistance gene *Lr16*. BMC Plant Biol 17:1-9

Kolmer J, Bernardo A, Bai G, Hayden M, Chao S (2018a) Adult plant leaf rust resistance derived from Toropi wheat is conditioned by *Lr78* and three minor QTL. Phytopathol 108:246-253

Kolmer J, Chao S, Brown‐Guedira G, Bansal U, Bariana H (2018b) Adult plant leaf rust resistance derived from the soft red winter wheat cultivar ‘Caldwell’maps to chromosome 3BS. Crop Sci 58:152-158

Kolmer JA, Su Z, Bernardo A, Bai G, Chao S (2018c) Mapping and characterization of the new adult plant leaf rust resistance gene *Lr77* derived from Santa Fe winter wheat. Theor Appl Genet 131:1553-1560

Kolmer J, Bernardo A, Bai G, Hayden M, Anderson J (2019) Thatcher wheat line RL6149 carries *Lr64* and a second leaf rust resistance gene on chromosome 1DS. Theor Appl Genet 132:2809-2814

Kolmer J, Bajgain P, Rouse M, Li J, Zhang P (2023) Mapping and characterization of the recessive leaf rust resistance gene *Lr83* on wheat chromosome arm 1DS. Theor Appl Genet 136:115

Kolodziej M, Singla J, Sánchez-Martín J, Zbinden H, Šimková H, Karafiátová M, Doležel J, Gronnier J, Poretti M, Glauser G, Zhu W, Köster P, Zipfel C, Wicker T, Krattinger SG, Keller B (2021) A membrane-bound ankyrin repeat protein confers race-specific leaf rust disease resistance in wheat. Nat Commun 12: 956.

Kumar S, Bhardwaj SC, Gangwar OP, Sharma A, Qureshi N, Kumaran VV, Khan H, Prasad P, Miah H, Singh GP, Sharma K, Verma H, Forrest KL, Trethowan RM, Bariana HS, Bansal UK (2021) *Lr80*: A new and widely effective source of leaf rust resistance of wheat for enhancing diversity of resistance among modern cultivars. Theor Appl Genet 134:849-858

Kuraparthy V, Sood S, See DR, Gill BS (2009) Development of a PCR assay and marker‐assisted transfer of leaf rust and stripe rust resistance genes *Lr57* and *Yr40* into hard red winter wheats. Crop Sci 49:120-126

Lagudah E, McFadden H, Singh R, Huerta-Espino J, Bariana H, Spielmeyer W (2006) Molecular genetic characterization of the *Lr34/Yr18* slow rusting resistance gene region in wheat. Theor Appl Genet 114:21-30

Lagudah ES, Krattinger SG, Herrera-Foessel S, Singh RP, Huerta-Espino J, Spielmeyer W, Brown-Guedira G, Selter LL, Keller B (2009) Gene-specific markers for the wheat gene *Lr34/Yr18/Pm38* which confers resistance to multiple fungal pathogens. Theor Appl Genet 119:889-898

Li C, Wang Z, Li C, Bowden R, Bai G, Li C, Li C, Su Z, Carver BF (2017) Mapping of quantitative trait loci for leaf rust resistance in the wheat population Ning7840× Clark. Plant Dis 101:1974-1979

Lin G, Chen H, Tian B, Sehgal SK, Singh L, Xie J, Rawat N, Juliana P, Singh N, Shrestha S, Wilson DL, Shult H, Lee H, Schoen AW, Tiwari VK, Singh RP, Guttieri MJ, Trick HN, Poland J, Bowden RL, Bai G, Gill B, Liu S (2022) Cloning of the broadly effective wheat leaf rust resistance gene *Lr42* transferred from *Aegilops tauschii*. Nat Commun 13:3044

Liu Y, Chen H, Li C, Zhang L, Shao M, Pang Y, Xu X, Bai G (2021) Development of diagnostic markers for a wheat leaf rust resistance gene *Lr42* using RNA-sequencing. Crop J 9:1357-1366

Megerssa SH, Ammar K, Acevedo M, Brown-Guedira G, Ward B, Degete AG, Randhawa MS, Sorrells ME (2020) Multiple-race stem rust resistance loci identified in durum wheat using genome-wide association mapping. Front Plant Sci 11:598509

Naz AA, Bungartz A, Serfling A, Kamruzzaman M, Schneider M, Wulff BB, Pillen K, Ballvora A, Oerke EC, Ordon F, Léon J (2021) *Lr21* diversity unveils footprints of wheat evolution and its new role in broad‐spectrum leaf rust resistance. Plant Cell Environ 44:3445-3458

Neelam K, Brown-Guedira G, Huang L (2013) Development and validation of a breeder-friendly KASPar marker for wheat leaf rust resistance locus *Lr21*. Mol Breed 31:233-237

Nsabiyera V, Qureshi N, Bariana HS, Wong D, Forrest KL, Hayden MJ, Bansal UK (2016) Molecular markers for adult plant leaf rust resistance gene *Lr48* in wheat. Mol Breed 36:1-9

Qiu L, Wang H, Li Y, Wang W, Liu Y, Mu J, Geng M, Guo W, Hu Z, Ma J, Sun Q, Xie C (2020) Fine mapping of the wheat leaf rust resistance gene *LrLC10* (*Lr13*) and validation of its co-segregation markers. Front Plant Sci 11:470

Qureshi N, Bariana H, Kumran VV, Muruga S, Forrest KL, Hayden MJ, Bansal U (2018) A new leaf rust resistance gene *Lr79* mapped in chromosome 3BL from the durum wheat landrace Aus26582. Theor Appl Genet 131:1091-1098

Rani K, Raghu B, Jha S, Agarwal P, Mallick N, Niranjana M, Sharma J, Singh A, Sharma N, Rajkumar S, Tomar SMS, Vinod (2020) A novel leaf rust resistance gene introgressed from *Aegilops markgrafii* maps on chromosome arm 2AS of wheat. Theor Appl Genet 133:2685-2694

Rauf Y, Lan C, Randhawa M, Singh RP, Huerta‐Espino J, Anderson JA (2022) Quantitative trait loci mapping reveals the complexity of adult plant resistance to leaf rust in spring wheat ‘Copio’. Crop Sci 62:1037-1050

Rosa SB, Zanella CM, Hiebert CW, Brûlé-Babel AL, Randhawa HS, Shorter S, Boyd LA, McCallum BD (2019) Genetic characterization of leaf and stripe rust resistance in the Brazilian wheat cultivar Toropi. Phytopathol 109:1760-1768

Sapkota S, Mergoum M, Kumar A, Fiedler JD, Johnson J, Bland D, Lopez B, Sutton S, Ghimire B, Buck J, Chen Z, Harrison S (2020) A novel adult plant leaf rust resistance gene *Lr2K38* mapped on wheat chromosome 1AL. Plant Genome 13:e20061

Sharma JS, McCartney CA, McCallum BD, Hiebert CW (2023) Fine mapping and marker development for the wheat leaf rust resistance gene *Lr32*. Genes Genom Genet 13: jkac274

Thind AK, Wicker T, Šimková H, Fossati D, Moullet O, Brabant C, Vrána J, Doležel J, Krattinger SG (2017) Rapid cloning of genes in hexaploid wheat using cultivar-specific long-range chromosome assembly. Nat Biotechnol 35:793-796

Wu J, Wang Q, Kang Z, Liu S, Li H, Mu J, Dai M, Han D, Zeng Q, Chen X (2017) Development and validation of KASP-SNP markers for QTL underlying resistance to stripe rust in common wheat cultivar P10057. Plant Dis 101:2079-2087

Xu X, Kolmer J, Li G, Tan C, Carver BF, Bian R, Bernardo A, Bai G (2022) Identification and characterization of the novel leaf rust resistance gene *Lr81* in wheat. Theor Appl Genet 135:2725-2734

Xu X, Li G, Bai G, Bernardo A, Carver BF, St. Amand P, Bian R (2021) Characterization of an incomplete leaf rust resistance gene on chromosome 1RS and development of KASP markers for *Lr47* in wheat. Phytopathol 111:649-658

Xue S, Kolmer JA, Wang S, Yan L (2018) Mapping of leaf rust resistance genes and molecular characterization of the 2NS/2AS translocation in the wheat cultivar Jagger. Genes Genom Genet 8:2059-2065

Yan X, Li M, Zhang P, Yin G, Zhang H, Gebrewahid TW, Zhang J, Dong L, Liu D, Liu Z, Li Z (2021) High-temperature wheat leaf rust resistance gene *Lr13* exhibits pleiotropic effects on hybrid necrosis. Mol Plant 14:1029-1032

Zhang Q, Wei W, Zuansun X, Zhang S, Wang C, Liu N, Qiu L, Wang W, Guo W, Ma J, Peng H, Hu Z, Sun Q, Xie C (2021) Fine mapping of the leaf rust resistance gene *Lr65* in spelt Wheat ‘Altgold’. Front Plant Sci 12:666921
