# Supplementary material for: Wheat improvement through advances in single nucleotide polymorphism (SNP) detection and genotyping with a special emphasis on rust resistance
Source: Theor Appl Genet. 2024 Sep 16;137(10):224. doi: 10.1007/s00122-024-04730-w (PMC11405505; doi:10.1007/s00122-024-04730-w)
Supplement: Supplementary file 2 — Supplementary file2 (DOCX 32 KB) [file 122_2024_4730_MOESM2_ESM.docx]

**Supplementary table 1b: Role of SNPs in mapping and selection of known stem rust resistance genes**

| Genes | Types | Chromosome | SNPs (for selection) | Assay type | Flanking/linked SNPs | Arrays/  marker source | Mapping population/germplasm | References |
| --- | --- | --- | --- | --- | --- | --- | --- | --- |
| *Sr5*/  QSr.ace-6D1 | ASR | 6D | - | - | IWB262  IWB6902 | 90K SNP array | 157 RILs of LMPG-6/PI 362698-1 cross | Zurn et al. (2018) |
| *Sr6* | ASR | 2D | - | - | S2D_61759932 | GBS | 270 winter wheat genotypes, 60 genotypes from e 2015 F_3:7_ nurseries (Nebraska Nursery) | (Mourad et al. 2018) |
| *Sr7a* | ASR | 4A | 7126 | KASP | 1067 to 7126 | 9K SNP array | 164 BC_1_F_2_ families of Jagger/LMPG-6 cross | Turner et al. (2016) |
| *Sr8A/**Sr8155B1/**QSr.spa-6A/* *QSr.ace-6A* | ASR | 6A | - | - | IAAV8377  wPt-5652  wsnp_Ra_c3996_7334169 | 90K SNP array  DArT | 66DH lines of A9919-BY5C/Strongfield cross | Babiker et al. (2017a); Hiebert et al. (2017); Kumar et al. (2021) |
| Sr8a/QSr.ace-6A | ASR | 6A | NB-LRR3 | KASP | IWB49090  IWB67416 | 90K SNP array | 157 RILs of LMPG-6/PI 362698-1 cross | Zurn et al. (2018) |
| *Sr8155B1* | ASR | 6A | KASP 6AS_IWB10558 | KASP | IWB64918  IWB43809  IWB10558 | 90K SNP array | 152 F_2_ plants and 143 BC_1_F_2_  families of Rusty/8155-B1 cross | Nirmala et al. (2017) |
| *Sr883-2B* | ASR | 2B | fcp716  fcp717 | STARP | IWB56465  IWB55767 | 90K SNP array | 190 RILs of Rusty/PI 193883 cross | Sharma et al. (2019) |
| *Sr9h/ QSr.abr-2BL.1* | ASR | 2B | IWB51807  IWB4357  IWB72651  IWB56162  IWB7850 | KASP | IWB72650  IWB72651 | 90K SNP array | 121 DH lines and 124 RILs of LMPG-6/CItr 4311 cross | Babiker et al. (2016) |
| *Sr9h* | ASR | 2B | - | - | XIWA543-HRM | 2B specific HRM markers from 9K array | 247 F_2:3_ lines of Matlabas/Line 37-07 cross | Wessels et al. (2019) |
| *Sr11* | ASR | 6B | KASP_6BL_IWB10724 KASP_6BL_IWB72471 |  | IWB73072/ IWB10724 | 90K SNP array | 149 RILs of Gabo 56/Chinese Spring cross | Nirmala et al. (2016) |
| *Sr12* | ASR | 3B | NB-LRR3 | KASP | IWA6086   IWA4613 | 9K SNP array | 94 lines of RL90/RL6058 cross; 137 DH lines of Chinese Spring/RL6058 cross; 160 RILs of McNeal/Thatcher cross | Hiebert et al. (2016) |
| Sr12/QSr.ace-3B | ASR | 3B | NB-LRR3 | KASP | IWB68368  IWA4847 | 90K SNP array | 157 RILs of LMPG-6/PI362698-1 cross | Zurn et al. (2018) |
| *Sr13* | ASR | 6A | T2200C | dCAPS with HhaI | - | BAC sequencing | F_2_ plants derived from Kronos / Rusty cross | Zhang et al. (2017) |
| *Sr13/**Sr883-6A/* | ASR | 6A | rwgsnp7, rwgsnp37 rwgsnp38, rwgsnp39,  rwgsnp40, KASPSr13 | STARP  KASP | IWB3057   barc104 | 90K SNP array | 190 RILs of Rusty /PI 193883 cross | Gill et al. (2021); Sharma et al. (2019) |
| *Sr14/**QSr.spa-1B* | ASR | 1B | - | - | wPt-2389   u_c8767_1245 | 90K SNP array  DArT | 66DH lines of A9919-BY5C/Strongfield cross | Kumar et al. (2021) |
| *Sr15/**QSr.abr-7AL* | ASR | 7A | KASP_IWB30995 | KASP | IWB46162/ IWB28967 | 90K SNP array KASP | 185 RILs and 192 DH lines of LMPG-6/PI374670 cross | Babiker et al. (2015); Gao et al. (2019) |
| *Sr16/*QSr.ace-2B | ASR | 2B | - | - | IWB2335  IWB23589 | 90K SNP array | 157 RILs of LMPG-6/PI362698-1 cross | Zurn et al. (2018) |
| Sr17/ QSr.cnl-7B | FR | 7B | - | - | S7B_677752911  S7B_688049535 | GBS | 224 RILs of DAKIYE/Reichenbachii | Megerssa et al. (2022) |
| Sr21 | ASR | 2A^m^ | SNPC1228W | CAPS | FD527726  EX594406 | Sequence based markers | 734 F_2_ plants of PI 272557/ DV92 and 2850 F_2_ plants of PI272557/G3116 cross | Chen et al. (2015); Chen et al. (2018b) |
| *Sr22b /SrTm5* | ASR | 7A^m^ | TM5TF2R2  pkw4974 | INDEL  CAPS | IWB25012  IWB44281 IWB40527  pkw4995  pkw4999 | 90K SNP array  Map based cloning | 1132 F_2:3_ populations derived from G3116 / PI306540 and PI272557 / PI306540 crosses | Chen et al. (2018a); Luo et al. (2022) |
| *Sr25* | APR | 7D | CAP7_c2912_1387634 | KASP | Ex_c5884_10325223 JD_c6624_7769357 CAP7_c2912_1387634  Ex_c5884_10325223 | 9K SNP array | 189 Wheat breeding lines from 5^th^ SRRSN | Yu et al. (2017) |
| *Sr26* | ASR | 6A | sunKASP_224 sunKASP_225 | KASP | sunKASP_216 sunKASP_218 sunKASP_224 sunKASP_225 | GBS  90K SNP array | 130 RILs of Aus27969/Avocet S cross | Qureshi et al. (2018) |
| *Sr28/**QSr.abr-2BL.1* | ASR | 2B | KASP_IWB1208 | KASP | IWB1208  IWB23232  IWB57292 | 90K SNP array | 140RILs and 138DH lines of LMPG-6/ PI177906 cross | Babiker et al. (2017b) |
| *Sr42* | ASR | 6D | TP43472 | KASP | IWB15852, IWB31561, IWB36391, WCSS1_ 6DS_2123217-1527 (TP43472), WCSS1_6DS_ 2061773-14642 (TP93838) | GBS  90K SNP array | 94 F_2:3_ population of LMPG-6/PI595667 cross  108 DH lines of PI410954 | Gao et al. (2015) |
| *Sr48* | ASR  ASR | 2D | sunKASP_239 | KASP | sunKASP_239 | DArTSeq | 172 F_5:7_ RIL population of Arina/Cezanne cross | Nsabiyera et al. (2023) |
|  |  | 2A | - | - | IWA1202–IWA574  IWA3819- IWA1496 | 9K SNP array | 1411 hexaploid winter wheat accessions | Mihalyov et al. (2017) |
| *Sr56/**QSr.spa-5B* | APR | 5B |  |  | Tdurum_contig28868_84 and BobWhite_c7818_278 | 90K SNP array  DArT | 66DH lines of A9919-BY5C / Strong field cross | Kumar et al. (2021) |
| *Sr56* | APR | 5B | - | - | IWA6902  (sun469) | 9K SNP array | 178 RILs of Arina/Yitpi cross | Bansal et al. (2014) |
| *Sr60* | ASR | 5A^m^ | Sr60F2/R2  DK722976F5R5 | CAPS | GH724575-CJ942731 | 90K SNP array | F_2:3_ populations derived from G3116/PI306540 and PI 272557/PI306540 crosses | Chen et al. (2018a); Chen et al. (2020) |
| *Sr63/ QSrGH.cs-2AL* | APR | 2A | KASP_32429 | KASP | IWB6412  IWB58452 | 90K array | 210 RILs of GH/Bansi cross | Mago et al. (2022) |
| *SrTm4* | ASR | 2A^m^ | - | - | CS4211-130K1519 | RNA-seq data | F_2:3_ populations derived from G3116/ PI306540 and PI272557 /PI306540 crosses | Li et al. (2023) |
| *SrKN* | ASR | 2B | - | - | pku4856F2R2-pku4917F3R3 | 90K SNP array  Exome-capture | F_2:3_ populations and/F_4_/F_5_ progenies of Kronos sr13 mutant line T4-3102/Rusty cross  23 accessions of *T. turgidum* ssp. *durum* and 16 of *T. aestivum* | Li et al. (2021) |
| *SrWLR* | ASR | 2B | - | - | IWA6121, IWA6122, IWA7620, IWA8295, IWA8362 | 9K SNP array | 166 F_2_ progenies of LMPG-6/PI626573 cross | Zurn et al. (2014) |
| *SrH* |  | 2B | cim117 | KASP | cim109, cim114  cim117 | 35K SNP array  KASP | 148 RILs of Cacuke/Huhwa cross  198 RILs of Cacuke/Yaye cross | Randhawa et al. (2018) |
| *SrY* |  | 2B | - | - | cim109, cim116  IWB45932 |  |  |  |

**References**

Babiker E, Gordon T, Chao S, Newcomb M, Rouse MN, Jin Y, Wanyera R, Acevedo M, Brown-Guedira G, Williamson S, Bonman JM (2015) Mapping resistance to the Ug99 race group of the stem rust pathogen in a spring wheat landrace. Theor Appl Genet 128:605-612

Babiker E, Gordon T, Chao S, Rouse M, Wanyera R, Newcomb M, Brown-Guedira G, Pretorius Z, Bonman J (2016) Genetic mapping of resistance to the Ug99 race group of *Puccinia graminis* f. sp. *tritici* in a spring wheat landrace CItr 4311. Theor Appl Genet 129:2161-2170

Babiker E, Gordon T, Bonman J, Chao S, Rouse M, Jin Y, Newcomb M, Wanyera R, Bhavani S (2017a) Genetic loci conditioning adult plant resistance to the Ug99 race group and seedling resistance to races TRTTF and TTTTF of the stem rust pathogen in wheat landrace CItr 15026. Plant Dis 101:496-501

Babiker E, Gordon T, Chao S, Rouse M, Wanyera R, Acevedo M, Brown-Guedira G, Bonman J (2017b) Molecular mapping of stem rust resistance loci effective against the Ug99 race group of the stem rust pathogen and validation of a single nucleotide polymorphism marker linked to stem rust resistance gene *Sr28*. Phytopathol 107:208-215

Bansal U, Bariana H, Wong D, Randhawa M, Wicker T, Hayden M, Keller B (2014) Molecular mapping of an adult plant stem rust resistance gene *Sr56* in winter wheat cultivar Arina. Theor Appl Genet 127:1441-1448

Chen S, Rouse MN, Zhang W, Jin Y, Akhunov E, Wei Y, Dubcovsky J (2015) Fine mapping and characterization of *Sr21*, a temperature-sensitive diploid wheat resistance gene effective against the *Puccinia graminis* f. sp. *tritici* Ug99 race group. Theor Appl Genet 128:645-656

Chen S, Guo Y, Briggs J, Dubach F, Chao S, Zhang W, Rouse MN, Dubcovsky J (2018a) Mapping and characterization of wheat stem rust resistance genes *SrTm5* and *Sr60* from *Triticum monococcum*. Theor Appl Genet 131:625-635

Chen S, Zhang W, Bolus S, Rouse MN, Dubcovsky J (2018b) Identification and characterization of wheat stem rust resistance gene *Sr21* effective against the Ug99 race group at high temperature. PLoS Genet 14:e1007287

Chen S, Rouse MN, Zhang W, Zhang X, Guo Y, Briggs J, Dubcovsky J (2020) Wheat gene *Sr60* encodes a protein with two putative kinase domains that confers resistance to stem rust. New Phytol 225:948-959

Gao L, Kielsmeier-Cook J, Bajgain P, Zhang X, Chao S, Rouse MN, Anderson JA (2015) Development of genotyping by sequencing (GBS)-and array-derived SNP markers for stem rust resistance gene *Sr42*. Mol Breed 35:1-13

Gao L, Babiker E, Nava I, Nirmala J, Bedo Z, Lang L, Chao S, Gale S, Jin Y, Anderson JA, Bansal U, Park RF, Rouse MN, Bonman JM, Bariana H (2019) Temperature‐sensitive wheat stem rust resistance gene *Sr15* is effective against *Puccinia graminis* f. sp. *tritici* race TTKSK. Plant Pathol 68:143-151

Gill BK, Klindworth DL, Rouse MN, Zhang J, Zhang Q, Sharma JS, Chu C, Long Y, Chao S, Olivera PD, Friesen TL, Zhong S, Jin Y, Faris JD, Fiedler JD, Elias EM, Liu S, Cai X, Xu SS (2021) Function and evolution of allelic variations of *Sr13* conferring resistance to stem rust in tetraploid wheat (*Triticum turgidum* L.). Plant J 106:1674-1691

Hiebert CW, Kolmer JA, McCartney CA, Briggs J, Fetch T, Bariana H, Choulet F, Rouse MN, Spielmeyer W (2016) Major gene for field stem rust resistance co-locates with resistance gene *Sr12* in ‘Thatcher’wheat. PLoS One 11:e0157029

Hiebert CW, Rouse MN, Nirmala J, Fetch T (2017) Genetic mapping of stem rust resistance to *Puccinia graminis* f. sp. *tritici* race TRTTF in the Canadian wheat cultivar Harvest. Phytopathol 107:192-197

Kumar S, Fetch TG, Knox RE, Singh AK, Clarke JM, Depauw RM, Cuthbert RD, Campbell HL, Singh D, Bhavani S, Pozniak CJ, Meyer B, Clarke FR (2021) Mapping of Ug99 stem rust resistance in Canadian durum wheat. Can J Plant Pathol 43:599-611

Li H, Hua L, Rouse MN, Li T, Pang S, Bai S, Shen T, Luo J, Li H, Zhang W, Wang X, Dubcovsky J, Chen S (2021) Mapping and characterization of a wheat stem rust resistance gene in durum wheat “Kronos”. Front Plant Sci 12:751398

Li H, Luo J, Zhang W, Hua L, Li K, Wang J, Xu B, Yang C, Wang G, Rouse MN, Dubcovsky J, Chen S (2023) High-resolution mapping of *SrTm4*, a recessive resistance gene to wheat stem rust. Theor Appl Genet 136:120

Luo J, Rouse MN, Hua L, Li H, Li B, Li T, Zhang W, Gao C, Wang Y, Dubcovsky J, Chen S (2022) Identification and characterization of *Sr22b*, a new allele of the wheat stem rust resistance gene *Sr22* effective against the Ug99 race group. Plant Biotechnol J 20:554-563

Mago R, Chen C, Xia X, Whan A, Forrest K, Basnet BR, Perera G, Chandramohan S,

Randhawa M, Hayden M, Bansal U, Huerta-Espino J, Singh RP, Bariana H, Lagudah E (2022) Adult plant stem rust resistance in durum wheat Glossy Huguenot: mapping, marker development and validation. Theor Appl Genet 135:1541-1550

Megerssa SH, Ammar K, Acevedo M, Bergstrom GC, Dreisigacker S, Randhawa M, Brown-Guedira G, Ward B, Sorrells ME (2022) QTL mapping of seedling and field resistance to stem rust in DAKIYE/Reichenbachii durum wheat population. PLoS One17:e0273993

Mihalyov PD, Nichols VA, Bulli P, Rouse MN, Pumphrey MO (2017) Multi‐locus mixed model analysis of stem rust resistance in winter wheat. Plant Genome <https://doi.org/10.3835/plantgenome2017.01.0001>

Mourad AM, Sallam A, Belamkar V, Wegulo S, Bowden R, Jin Y, Mahdy E, Bakheit B, El-Wafaa AA, Poland J, Baenziger PS (2018) Genome-wide association study for identification and validation of novel SNP markers for *Sr6* stem rust resistance gene in bread wheat. Front Plant Sci 9:380

Nirmala J, Chao S, Olivera P, Babiker EM, Abeyo B, Tadesse Z, Imtiaz M, Talbert L, Blake NK, Akhunov E, Pumphrey MO, Jin Y, Rouse MN (2016) Markers linked to wheat stem rust resistance gene *Sr11* effective to *Puccinia graminis* f. sp. *tritici* race TKTTF. Phytopathol 106:1352-1358

Nirmala J, Saini J, Newcomb M, Olivera P, Gale S, Klindworth D, Elias E, Talbert L, Chao S, Faris J, Xu S, Jin Y, Rouse MN (2017) Discovery of a novel stem rust resistance allele in durum wheat that exhibits differential reactions to Ug99 isolates. Genes Genom Genet 7:3481-3490

Nsabiyera V, Qureshi N, Li J, Randhawa M, Zhang P, Forrest K, Bansal U, Bariana H (2023) Relocation of *Sr48* to Chromosome 2D Using an Alternative Mapping Population and Development of a Closely Linked Marker Using Diverse Molecular Technologies. Plants 12:1601

Qureshi N, Kandiah P, Gessese MK, Nsabiyera V, Wells V, Babu P, Wong D, Hayden M, Bariana H, Bansal U (2018) Development of co-dominant KASP markers co-segregating with Ug99 effective stem rust resistance gene *Sr26* in wheat. Mol Breed 38:1-9

Randhawa MS, Singh RP, Dreisigacker S, Bhavani S, Huerta-Espino J, Rouse MN, Nirmala J, Sandoval-Sanchez M (2018) Identification and validation of a common stem rust resistance locus in two bi-parental populations. Front Plant Sci 9:1788

Sharma JS, Zhang Q, Rouse MN, Klindworth DL, Friesen TL, Long Y, Olivera PD, Jin Y, McClean PE, Xu SS, Faris JD (2019) Mapping and characterization of two stem rust resistance genes derived from cultivated emmer wheat accession PI 193883. Theor Appl Genet 132:3177-3189

Turner MK, Jin Y, Rouse MN, Anderson JA (2016) Stem rust resistance in ‘Jagger’winter wheat. Crop Sci 56:1719-1725

Wessels E, Prins R, Boshoff WH, Zurn JD, Acevedo M, Pretorius ZA (2019) Mapping a resistance gene to *Puccinia graminis* f. sp. *tritici* in the bread wheat cultivar ‘Matlabas’. Plant Dis 103:2337-2344

Yu L-X, Chao S, Singh RP, Sorrells ME (2017) Identification and validation of single nucleotide polymorphic markers linked to Ug99 stem rust resistance in spring wheat. PLoS One 12:e0171963

Zhang W, Chen S, Abate Z, Nirmala J, Rouse MN, Dubcovsky J (2017) Identification and characterization of *Sr13*, a tetraploid wheat gene that confers resistance to the Ug99 stem rust race group. Proc Natl Acad Sci USA 114:E9483-E9492

Zurn JD, Newcomb M, Rouse MN, Jin Y, Chao S, Sthapit J, See DR, Wanyera R, Njau P, Bonman JM, Brueggeman R,Acevedo M (2014) High-density mapping of a resistance gene to Ug99 from the Iranian landrace PI 626573. Mol Breed 34:871-881

Zurn JD, Rouse MN, Chao S, Aoun M, Macharia G, Hiebert CW, Pretorius ZA, Bonman JM, Acevedo M (2018) Dissection of the multigenic wheat stem rust resistance present in the Montenegrin spring wheat accession PI 362698. BMC Genomics 19:1-11
