# Supplementary material for: Wheat improvement through advances in single nucleotide polymorphism (SNP) detection and genotyping with a special emphasis on rust resistance
Source: Theor Appl Genet. 2024 Sep 16;137(10):224. doi: 10.1007/s00122-024-04730-w (PMC11405505; doi:10.1007/s00122-024-04730-w)
Supplement: Supplementary file 3 — Supplementary file3 (DOCX 30 KB) [file 122_2024_4730_MOESM3_ESM.docx]

**Supplementary table 1c: Role of SNPs in mapping and selection of known stripe rust resistance genes**

| Genes | Types | Chromosome | SNPs (for selection) | Assay type | Flanking/  linked SNPs | Arrays/marker source | Mapping population/ germplasm | References |
| --- | --- | --- | --- | --- | --- | --- | --- | --- |
| *Yr5* | ASR | 2B | *Yr5*STS7/8_CAPS  STS9+STS10- DpnII digestion | CAPS | STS7/8 | CAPS | 93 BC_7_F_3_ individuals of Yr5/6*AVS cross | Murphy et al. (2009) |
|  |  |  | *Yr5*_KASP | KASP | - | MutRenSeq | - | Marchal et al. (2018) |
|  |  |  |  | KASP | IWA6121  IWA4096 | 9K array | RILs of WA8149/ S0900317and  WA8149 / S0900163 crosses | Naruoka et al. (2016) |
| *Yr15* | ASR | 1B | R5, R8 | KASP | R5, R11 to R8 | BSR-seq | 196 F_2_ individuals from NILs AvocetS+Yr15/Avocet S | Ramirez‐Gonzalez et al. (2015) |
| *Yr17* *(YrMM58/ YrHY1)* | ASR | 2A | - | - | WGGB191  WGGB196 | BSR-Seq | >200 F_2:3_ populations derived from Nongda 399/Mengmai 58 and Nongda 399/Huaiyang 1 crosses | Wang et al. (2017) |
| *Yr26* | ASR | 1B | CM1461  CM501  WRS467 | KASP | WRS435  WRS312 | RNA Seq  90K SNP array  660K SNP array | 2341 F_2_ individuals and 156 RILs from AvS/92R137 cross  273 RILs from YM5/92R137 cross  13,128 F_2_ plants, 240 F_2:3_ lines and 1034 RILs from NIL-S/NIL-R | Wu et al. (2018b) |
| *Yr29* | APR | 1B | - | - | SNPLr46G22 | 90 K SNP array  tGBS | 130 RILs derived from Aus27969/Avocet ‘S’cross | Pakeerathan et al. (2019) |
| Yr29/Lr46/  QYrqin.nwafu-1BL/ QYrcw.nwafu-1BL | APR | 1B | AX-94509279  AX-94673495 | KASP | AX-95139868  AX-94885318 | 1. 35K SNP array 660K SNP array | 165 RILs of Avocet S/QN142 cross | Zeng et al. (2019a) |
|  |  |  | csLV46G22 | CAPS | AX-95026093  AX-94701609 | 1. 35K SNP array | 128 RILs of MX69/CW86 cross | Zeng et al. (2019b) |
| *Yr30/Sr2/*  *QYr.nwafu-3BS.2* | APR | 3B | - | - | AX-111487728  AX-109919508 | 1. 55K SNP array | 186 RILs of Chinese landrace Mingxian169/P9936 cross | Huang et al. (2019) |
| *Yr66 (YrVL1)* | ASR | 3D | - | - | KASP 48179  KASP 18087 | 1. 90K SNP array | 100 F_2:3_ families of VL Gehun 892/Westonia cross | Bariana et al. (2022) |
| *Yr67 (YrVL2)* | ASR | 7B | - | - | KASP_37096 KASP_2239 | 1. 90K SNP array | 100 F_2:3_ families of VL Gehun 892/Westonia cross | Bariana et al. (2022) |
| *Yr71/YrSA3* | APR | 3D | KASP_16434  KASP_17207  KASP_20836 | KASP | KASP_16434,  KASP_17207  KASP_20836 | 1. 90K SNP array | 123 RILs of SA65/SA67 cross | Bariana et al. (2016) |
| *Yr78/*  *QYr.ucw-6B/*  *QYr.sun-6B/*  *QYr.wgp-6BS.1* | APR | 6B | IWA7257 | KASP | IWA7257 | 9K SNP array  KASP assay | F_2_ lines of PI519805/Avocet ‘S’ cross  10 different F_2_ biparental mapping populations developed by crossing resistant donors with Avocet | Dong et al. (2017); Santra et al. (2008) |
|  |  |  | CDM158 CDM160-2 | KASP | TraesCS6B02G116200 TraesCS6B02G118000 | Exome capture Pangenome | 3,062 F_3_ plants from selected 25 F_2_ plants from PI519805 / Avocet ‘S’ cross | Dang et al. (2022) |
| *Yr82* | APR | 3B | sun KASP_300  KASP_8775 | KASP | KASP_13376  KASP_8775 | 90K SNP array targeted GBS (tGBS) | 130 RILs derived from Aus27969/Avocet ‘S’cross | Pakeerathan et al. (2019) |
| *YrAs2388* | APR | 4D | HTM3g  KASP-E5  KASP-E6 | CAPS  KASP | Xsdauw2a  Xsdauw3a | 10K SNP array  Map-based cloning using fosmid library | Bulk segregants from 4205 F_3_ plants from three F_2_ populations (PI511383/PI486274) (CIae9/PI560536) (PI511384/AS87) | Hu et al. (2021); Zhang et al. (2019) |
| *YrKU* | ASR | 7B | - | - | 1070196 F 0-47:A>G 5324909 F 0-54:T>C | DArT-Seq GBS | 148 BC_1_F_5_ lines of Apav/KU3067 cross | Zhang et al. (2022) |
| *YrH62* | APR | 1B | AX-109352427  AX-109862469 | KASP | AX-109352427  AX-109862469 | 90 K SNP array 660 K SNP array | 167 F_2:3_ population of Avocet S/Line 03031-1-5 H62 cross | Wu et al. (2018a) |
| *Yr041133* | ASR | 7B | - | - | Xicst133  Xicst338 | BSR-Seq (SSRs from SNPs) | 176 RILs of Qingxinmai/041133 cross | Li et al. (2022) |
| *QYrXN3517-1BL* | APR | 1B | nwafu.a5 | Allele specific quantitative PCR (AQP) | 16 k-2430  16 k-2433 | 660K array  GenoBaits  16K SNP array BSE-Seq | 161 F_6_ RIL population of Avocet S (AvS)/XN3517 cross | Huang et al. (2023) |
| *YrZH22* | APR | 4B | WGGB133  WGGB119 | SNP | WGGB133  WGGB146 | BSR-Seq | RILs of Mingxian 169/Zhoumai 22 cross | Wang et al. (2017) |

**References**

Bariana H, Forrest K, Qureshi N, Miah H, Hayden M, Bansal U (2016) Adult plant stripe rust resistance gene *Yr71* maps close to *Lr24* in chromosome 3D of common wheat. Mol Breed 36:1-10

Bariana H, Kant L, Qureshi N, Forrest K, Miah H, Bansal U (2022) Identification and characterisation of stripe rust resistance genes *Yr66* and *Yr67* in wheat cultivar VL Gehun 892. Agronomy 12:318

Dang C, Zhang J, Dubcovsky J (2022) High‐resolution mapping of *Yr78*, an adult plant resistance gene to wheat stripe rust. Plant Genome 15:e20212

Dong Z, Hegarty JM, Zhang J, Zhang W, Chao S, Chen X, Zhou Y, Dubcovsky J (2017) Validation and characterization of a QTL for adult plant resistance to stripe rust on wheat chromosome arm 6BS (*Yr78*). Theor Appl Genet 130:2127-2137

Hu Y, Huang X, Wang F, He Y, Feng L, Jiang B, Hao M, Ning S, Yuan Z, Wu J, Zhang L, Wu B, Liu D, Huang L (2021) Development and validation of gene-specific KASP markers for *YrAS2388R* conferring stripe rust resistance in wheat. Euphytica 217:1-9

Huang S, Wu J, Wang X, Mu J, Xu Z, Zeng Q, Liu S, Wang Q, Kang Z, Han D (2019) Utilization of the genomewide wheat 55K SNP array for genetic analysis of stripe rust resistance in common wheat line P9936. Phytopathol 109:819-827

Huang S, Zhang Y, Ren H, Zhang X, Yu R, Liu S, Zeng Q, Wang Q, Yuan F, Singh RP, Bhavani S, Wu J, Han D, Kang Z (2023) High density mapping of wheat stripe rust resistance gene *QYrXN3517-1BL* using QTL mapping, BSE-Seq and candidate gene analysis. Theor Appl Genet 136:39

Li Y, Lin R, Hu J, Shi X, Qiu D, Wu P, Goitom GH, Wang S, Zhang H, Yang L, Liu H, Wu Q, Xie J, Zhou Y, Liu Z, Li H (2022) Mapping of wheat stripe rust resistance gene *Yr041133* by BSR-Seq analysis. Crop J 10:447-455

Marchal C, Zhang J, Zhang P, Fenwick P, Steuernagel B, Adamski NM, Boyd L, McIntosh R, Wulff BBH, Berry S, Lagudah E, Uauy C (2018) BED-domain-containing immune receptors confer diverse resistance spectra to yellow rust. Nat Plants 4:662-668

Murphy LR, Santra D, Kidwell K, Yan G, Chen X, Campbell KG (2009) Linkage maps of wheat stripe rust resistance genes *Yr5* and *Yr15* for use in marker‐assisted selection. Crop Sci 49:1786-1790

Naruoka Y, Ando K, Bulli P, Muleta KT, Rynearson S, Pumphrey MO (2016) Identification and validation of SNP markers linked to the stripe rust resistance gene *Yr5* in wheat. Crop Sci 56:3055-3065

Pakeerathan K, Bariana H, Qureshi N, Wong D, Hayden M, Bansal U (2019) Identification of a new source of stripe rust resistance *Yr82* in wheat. Theor Appl Genet 132:3169-3176

Ramirez‐Gonzalez RH, Segovia V, Bird N, Fenwick P, Holdgate S, Berry S, Jack P, Caccamo M, Uauy C (2015) RNA‐Seq bulked segregant analysis enables the identification of high‐resolution genetic markers for breeding in hexaploid wheat. Plant Biotechnol J 13:613-624

Santra D, Chen X, Santra M, Campbell K, Kidwell K (2008) Identification and mapping QTL for high-temperature adult-plant resistance to stripe rust in winter wheat (*Triticum aestivum* L.) cultivar ‘Stephens’. Theor Appl Genet 117:793-802

Wang Y, Xie J, Zhang H, Guo B, Ning S, Chen Y, Lu P, Wu Q, Li M, Zhang D, Guo G, Zhang Y, Liu D, Zou S, Tang J, Zhao H, Wang X, Li J, Yang W, Cao T, Yin G, Liu Z (2017) Mapping stripe rust resistance gene *YrZH22* in Chinese wheat cultivar Zhoumai 22 by bulked segregant RNA-Seq (BSR-Seq) and comparative genomics analyses. Theor Appl Genet 130:2191-2201

Wu J, Wang Q, Xu L, Chen X, Li B, Mu J, Zeng Q, Huang L, Han D, Kang Z (2018a) Combining SNP genotyping array with bulked segregant analysis to map a gene controlling adult-plant resistance to stripe rust in wheat line 03031-1-5 H62. Phytopathol 108:103-113

Wu J, Zeng Q, Wang Q, Liu S, Yu S, Mu J, Huang S, Sela H, Distelfeld A, Huang L, Han D, Kang Z (2018b) SNP-based pool genotyping and haplotype analysis accelerate fine-mapping of the wheat genomic region containing stripe rust resistance gene *Yr26*. Theor Appl Genet 131:1481-1496

Zeng Q, Wu J, Liu S, Chen X, Yuan F, Su P, Wang Q, Huang S, Mu J, Han D, Kang Z, Chen XM (2019a) Genome-wide mapping for stripe rust resistance loci in common wheat cultivar Qinnong 142. Plant Dis 103:439-447

Zeng Q, Wu J, Huang S, Yuan F, Liu S, Wang Q, Mu J, Yu S, Chen L, Han D, Kang Z (2019b) SNP-based linkage mapping for validation of adult plant stripe rust resistance QTL in common wheat cultivar Chakwal 86. Crop J 7:176-186

Zhang C, Huang L, Zhang H, Hao Q, Lyu B, Wang M, Epstein L, Liu M, Kou C, Qi J, Chen F, Li M, Gao G, Ni F, Zhang L, Hao M, Wang J, Chen X, Luo MC, Zheng Y, Wu J, Liu D, Fu D (2019) An ancestral NB-LRR with duplicated 3′ UTRs confers stripe rust resistance in wheat and barley. Nat Commun 10:4023

Zhang P, Lan C, Singh RP, Huerta-Espino J, Li Z, Lagudah E, Bhavani S (2022) Identification and characterization of resistance loci to wheat leaf rust and stripe rust in Afghan landrace “KU3067”. Front Plant Sci 13:894528
