# Supplementary material for: Wheat improvement through advances in single nucleotide polymorphism (SNP) detection and genotyping with a special emphasis on rust resistance
Source: Theor Appl Genet. 2024 Sep 16;137(10):224. doi: 10.1007/s00122-024-04730-w (PMC11405505; doi:10.1007/s00122-024-04730-w)
Supplement: Supplementary file 4 — Supplementary file4 (DOCX 36 KB) [file 122_2024_4730_MOESM4_ESM.docx]

**Supplementary table 2: SNP-based detection of novel QTLs for rust resistance through QTL mapping**

| Genes/QTL | Types | Chromosome | SNPs for selection | Flanking/linked SNPs | Arrays | Mapping population/germplasm | References |
| --- | --- | --- | --- | --- | --- | --- | --- |
| **Leaf rust** | | | | | | | |
| *QLr.hebau-2AL* | APR | 2A | - | wmc181-BS00057060_51 | 90K SNP array | 244 F_8_ RILs derived from Zhou 8425B/Chinese Spring cross | Zhang et al. (2017) |
| *QLr.hebau-3BS* | APR | 3B |  | BobWhite_c9711_71 Excalibur_c6330_1158 |  |  |  |
| *QLr.hebau-4AL* | APR | 4A | - | BobWhite_c15697_675-Excalibur_c2827_580 |  |  |  |
| *QLr.hebau-5BL* | APR | 5B |  | wsnp_Ex_c3175_5864335–BobWhite_c16916_658 |  |  |  |
| *QLr.hebau-5AL /*  *QYr.hebau-5AL* | ASR | 5A | AX_111493964  AX_11058589370 | AX_110909761  AX-110996595 | 55K SNP array | 144 F_6_ RILs derived from SW 8588/Thatcher cross | Zhang et al. (2019) |
| *QLr.hebau-3BL* | ASR | 3B | - | AX_111014259  AX_111534420 |  |  |  |
| *QLr.crc-5AL* | APR | 5A | Kwh425, Kwh426  Kwh428, Kwh429 | Excalibur_rep_c111129_125 Kukri_c12384_430 | 90K SNP array KASP | 168 DH lines from Toropi-6.4/Thatcher cross | Rosa et al. (2019) |
| *QLr.usw-2BS* | ASR | 2B | KASP_ Tdurum_ contig76118_145  KASP_ wsnp_Ex_ c18354_27181086 | Tdurum_contig76118_145   wsnp_Ex_c18354_27181086 | 90K SNP array | 200 RILs each from Gaza/ATRED #2, Arnacoris/ATRED #2, and Saragolla/ATRED #2crosses | Kthiri et al. (2019) |
| *QLr.usw-3B* | APR | 3B | KASP_Tdurum_contig33168_461   KASP_RAC875_rep_c82061_78 | Tdurum_contig33168_461   RAC875_rep_c82061_78 |  |  |  |
| *QLr.spa-4A* | APR | 4A | - | Ex_c70424_465 | 90K SNP array | Five DH populations: Carberry/ AC Cadillac, Carberry/Vesper, Vesper/Lillian, Vesper/Stettler and Stettler/Red Fife | Bokore et al. (2020) |
| *QLr.spa-5A* |  | 5A | - | BobWhite_c1387_798 |  |  |  |
| *QLr.ipbb-4A.2* | ASR | 4A | - | IAAV7104-Excalibur_c25699_113 | 20K SNP array | 98 RILs of PA/Par  (PamyatiAzieva/Paragon) cross | Genievskaya et al. (2020) |
| *QLr.ipbb-5B.2* | ASR | 5B | - | Kukri_rep_c98079_222-BS00075815_51 |  |  |  |
| *QLr.ipbb-7D.1* | ASR | 7D | - | Kukri_c16416_647-BS00062644_51 |  |  |  |
| *QLr.ipbb-4D.1* | ASR | 4D | - | TA020319-0161-BS00022436_51 |  |  |  |
| *QLr.ipbb-7A.3* | APR | 7A | - | Ra_c4601_2417-CAP7_c7296_88 |  |  |  |
| *QLr.cim-6BL* | APR | 6B | - | AX-95155193  AX-94562707 | 15K SNP array  35K SNP array  50K DArT array | 148 F_4_-derived F_5_ RILs from each of the Atred#2/Heller#1 and Atred#2/Dunkler; 148RILs from Bairds/Atred#2 cross | Lan et al. (2017); Li et al. (2020) |
| *QLr.cim-2BC* | APR | 2B | BA00290503.1  BA00817478 | AX-94522986  AX-94680690 |  |  |  |
| *QLr.stars-1RS* | ASR | 1B/1R | - | S1B_160486821  S1B_91879440 | GBS | RILs from CI17884/Bainong418 | Xu et al. (2021) |
| *QLr.cdl-2AL* | ASR | 2A | K-IWB6986 | BS00039406_51  BobWhite_c6356_87 | 90K SNP array | 120 RILs of Tc/411A cross | Kolmer and Rouse (2022) |
| *QLr.cdl-4BS* | ASR | 4B | K-IWB3541 | RAC875_c215_329  BobWhite_c46938_314 |  |  |  |
| *QLr.cdl.7AL* | ASR | 7A | K-IWB74437  K-IWB10682 | RFL_Contig2834_890  wsnp_Ku_c16022_24798741 |  |  |  |
| *QLr.cim-6BL* | APR | 6B | - | 1200827\|F\|0–33:A>G  1001678\|F\|0–37:A>G | DArTSeq  GBS | 148 BC_1_F_5_ lines of Apav/KU3067 | Zhang et al. (2022) |
| **Yellow rust** | | | | | | | |
| *Qyrnap.nwafu-2BS* | APR | 2B | - | 660K-AN21  660K-AN57 | 90 K SNP array  660 K SNP array | F_2:3_ lines and 175 F_2:6_ RILs of Avocet S/Napo 63 cross | Wu et al. (2017) |
| *QYr.nwafu*-*6BL* | APR | 6B | IWB71602  IWA4501 IWA55937 | IWB71602   IWB55937 | 90K SNP array | 150RILs of Mingxian 169/Friedrichswerther | Wu et al. (2018) |
| *QYrMa.wgp-1AS* | ASR | 1A | IWB54411, IWB12795  IWA5150 (KASP) | IWB54411  IWB57448 | GBS  90K SNP array | 156 RILs Avocet S/Madsen cross | Liu et al. (2018) |
| *QYrcen.nwafu-7BL* | APR | 7B | AX-94556751  AX-110366788 (KASP) | AX-94556751  AX-110366788 | 35K SNP array  660K SNP array | RILs of Mingxian 169/Centrum cross  F_2_ population of Xinong 979/Centrum cross | Mu et al. (2019a) |
| *QYr.nwafu-5AL* | APR | 5A | - | IWB5776 and IWB63558 | 90K array | 190 RILs of Xiaoyan81/ Xinong1376cross | (Mu et al. 2019b) |
| *QYr.nwafu-4AL* | APR | 4A | IWB6683-4A  IWB7070-4A  IWB42362-4A | IWB49885 and IWB6683 |  |  |  |
| *Qyr.nwafu-7BL* | APR | 7B | AX109317388  AX108987034 (KASP) | AX-108819274 – AX110470708 | 55KSNP array | 86 RILs of Mingxian169/CIMMYT derived line P9936 | Huang et al. (2019) |
| *QYr.hebau-5AL* | ASR | 5A | - | AX-110909761-AX_110957925 | 55K SNP array | 144 F_6_ RILs derived from SW 8588/Thatcher | Zhang et al. (2019) |
| *QYr.hebau-4BS* | ASR | 4B | - | AX-109867593 -AX_111641369 |  |  |  |
| *Qyr.hebau-6DS* | ASR | 6D | - | AX_109915375-AX_95175830 |  |  |  |
| *QYr.tamu-2B* | APR | 2B | IWB47487 IWB29391 IWB26631  (KASP) | IWB47487, IWB52095, IWB32069  IWB26631  IWB29391 | GBS  90K SNP array | 124 F_6_ RILs from TAM 112/TAM 111 cross | Yang et al. (2019) |
| *QYr.tamu-4A1.1* | APR | 4A | - | - |  |  |  |
| *QYr.tamu-4A1.2* | APR | 4A | - | - |  |  |  |
| QYrcw.nwafu-3BS | APR | 3B | AX-94679411 (A/G)  AX-94948985 (G/C) | AX-94437233- AX-95240191 | 35K SNP array | 128 RILs of Chakwal 86/Mingxian 169cross | Zeng et al. (2019a) |
| QYrqin.nwafu-2AL | APR | 2A | AX-94992915  AX-94757208 | AX-94655393 -AX-94895021 | 35K SNP array  660K SNP array | RILs of Avocet S/QN142cross | Zeng et al. (2019b) |
| QYrqin.nwafu-2BL | APR | 2B | AX-94588421  AX-94482474 | AX-94507002-AX-94562871 |  |  |  |
| QYrqin.nwafu-6BS | APR | 6B | AX-95134713  AX-94839736 | AX-9523501- AX95188301 |  |  |  |
| QYr.hebau-2AS | APR | 2A | - | AX-111018125-AX-110099691 | 55K array | 147 RILs of Fuyu 3/ Zhengzhou 5389 cross | Gebrewahid et al. (2020) |
| QYr.hebau-3AL/  QLr.hebau-3AL |  | 3A | - | AX-111087037-AX-110964176 |  |  |  |
| QYr.hebau-4BS |  | 4B | - | AX-108730259-AX-110740087 |  |  |  |
| QYr.hebau-5AL  QLr.hebau-5AL |  | 5A | - | AX-109446853- AX-111495975 |  |  |  |
| QYr.hebau-6DL |  | 6D | - | AX-108848475-AX-109273869 |  |  |  |
| QYr.hebau-7DL |  | 7D | - | AX-110938697- AX-111591751 |  |  |  |
| *Qyr.gaas.2A* | APR | 2A | - | cfd36- AX-110576889 | 55K SNP array | RILs derived from Mian 96–5/GX3 cross | Cheng et al. (2022) |
| *Qyr.gaas.6A* |  | 6A | - | AX-109558600-AX-109542604 |  |  |  |
| *QYr.cim-1BS* | APR | 1B | - | 4991863\|F\|0–7:C>A 1043386\|F\|0–14:A>G | DArTSeq GBS | 148 BC_1_F_5_ lines of Apav/KU3067 cross | Zhang et al. (2022) |
| *QYr.cim-2AL* | APR | 2A | - | 5370736\|F\|0–13:C>T and 1269847\|F\|0–7:T>A |  |  |  |
| *QYrXN3517-2BL* | ASR | 2B | - | 16 k-5738 to 16 k-5754 | 660K SNP array  16K SNP array BSE-Seq | 161 F_6_ RILs of Avocet S (AvS)/XN3517 cross | Huang et al. (2023) |
| **Stem rust** | | | | | | | |
| *7AL locus* | ASR | 7A | - | IWA3371  IWA4887 | 9K SNP array RadSeq, KASP | 196 F_2:3_ families of Col/Col-NS766 cross | Pujol et al. (2015) |
| *QSr.umn-6D* | APR | 6D | - | wsnp_BE445201D_Ta_1_1 to TP16823 | 9K SNP array GBS | 147 RILs of MN06113-8/RB07 cross | Bajgain et al. (2015) |
| *phenoF3_4TF* | ASR | 2U | - | Aeup1GBS11453, Aeup1GBS13266 | GBS | F_2:3_ population derived from PI542369/PI298905 cross | Edae et al. (2016) |
| *QSr.abr-2DS* | ASR | 2D | - | IWA989- IWB8481 | 90K SNP array KASP | 185 RILs and 192 DH lines from the LMPG-6/PI 374670 cross | Babiker et al. (2015) |
| *QSr.abr-6DS.1* | ASR | 6D | - | IWA2338  IWB262 | 90K SNP array | 140RILs and 138DH lines of LMPG-6/PI177906cross | Babiker et al. (2017) |
| *QSr.ace-7A2* | ASR | 7A | - | IWA1517  IWB9275 | 90 K SNP assay | 157 RILs of LMPG-6/PI362698-1 cross// | Zurn et al. (2018) |
| *QSr.ace-7A* | APR | 7A | - | WA8390 - IWA1805 (IWA8390) | 9K SNP array | 180 RILs of Rusty/PI192051-1 cross | Aoun et al. (2019) |
| *QSr.ipbb-6B.6* | ASR | 6B | - | wsnp_Ex_c12618_20079758-wsnp_Ex_c1249_2399894 | 20K SNP array | 98 RILs of cross PA/Par  (PamyatiAzieva/Paragon) cross | Genievskaya et al. (2020) |
| *QSr.ipbb-5B.1* | ASR | 5B | - | RAC875_rep_c114200_428-wsnp_RFL_Contig1548_762547 |  |  |  |
| *QSr.ipbb-5A.3* | ASR | 5A | - | BS00036851_51-Excalibur_c27357_146 |  |  |  |
| *QSr.ipbb-3D.1* | APR | 3D | - | Ra_c10284_405-Kukri_c5252_107 |  |  |  |
| *QSr.ipbb-5A.2* | APR | 5A | - | BS00089076_51-CAP11_c2623_196 |  |  |  |
| *QSr.iuy-7B* | APR | 7B | - | snp_7B_113572123 snp_7B_309747559 | GBS | 174 RILs of INIA Tero/ Baguette 13 cross; 107RILs of BR23//CEP19/PF85490/ 3/Baguette 13 cross | Baraibar et al. (2020) |
| *QSr.cdl-4AL* | ASR | 4A | - | Tdurum_contig42019_1714-BS00009680_51 | 90k SNP assay | RILs derived from LMPG-6 / CI 14275 cross | Kosgey et al. (2021) |
| *QSr.cdl-2BS.2* | APR | 2B | Excalibur_c7963_1722 (KASP) | Tdurum_contig54704_176 and BS00038820_51 |  |  |  |
| *QSr.cdl-3B.2* | APR | 3B | - | Excalibur_c57658_54, IAAV3838, RAC875_c10595_473, RAC875_c69_499 |  |  |  |
| *QSr.cdl-6A* | APR | 6A | - | BS00023627_51, IAAV3806, Excalibur_c60006_452 |  |  |  |
| *QSr-sparc-2B* | APR | 2B | kwh808 | Tdurum_contig29563_197   TA001263-0453 | SeqSNP  tGBS (SNPS selected from 90K array) | 227DH lines of  AAC Prevail/ BW961 cross | Bokore et al. (2021) |
| *QSr-sparc-7A* |  | 7A | kwh810, kwh811  kwh812 | Tdurum_contig59633_56   wsnp_Ra_c7112_12318340 |  |  |  |
| *QSr.spa-5B* | ASR | 5B |  | Tdurum_contig28868_84  BobWhite_c7818_278 | 90K SNP array  DArT | 66DH lines of A9919-BY5C/Strong field cross | Kumar et al. (2021) |
| *QSr.spa-4A* | ASR | 4A |  | D_GBF1XID01EMISV_148 |  |  |  |
| *QSr.umn-5D* | APR | 5D | - | Chr5D_422348015 | GBS | 176 F_4_-derived F_6_ RILs of Apav/Copio cross | Rauf et al. (2022) |
| *QSr_NCCR.6A.3* | ASR | 6A | - | IWB48751 | 90K SNP array DArT | Four RIL populations from  Latino/MG5323, Ciccio/Svevo, Cirillo/Neodur and NCCR multi-parent population (Neodur, Claudio, Colosseo and Rascon/2*Tarro) crosses | Marone et al. (2022) |
| *QSr_CxS.3A.1 and QSr_CxS.3A.2* | ASR | 3A | - | IWB66101 |  |  |  |
| *QSr_LxM.6B.1 and QSr_LxM.6B.2* | ASR | 6B | - | IWB60699 |  |  |  |
| QSr.cnl-3B | ASR | 3B | - | S3B_91123277 S3B_196992709  S3B_75830161 S3B_195726515 | GBS | 224 RILs of DAKIYE/Reichenbachii cross | Megerssa et al. (2022) |
| QSr.cnl-4B | APR | 4B | - | S4B_524068577  S4B_550731907 |  |  |  |

**References**

Aoun M, Kolmer JA, Rouse MN, Elias EM, Breiland M, Bulbula WD, Chao S, Acevedo M (2019) Mapping of novel leaf rust and stem rust resistance genes in the Portuguese durum wheat landrace PI 192051. Genes Genom Genet 9:2535-2547

Babiker E, Gordon T, Chao S, Newcomb M, Rouse MN, Jin Y, Wanyera R, Acevedo M, Brown-Guedira G, Williamson S, Bonman JM (2015) Mapping resistance to the Ug99 race group of the stem rust pathogen in a spring wheat landrace. Theor Appl Genet 128:605-612

Babiker E, Gordon T, Chao S, Rouse M, Wanyera R, Acevedo M, Brown-Guedira G, Bonman J (2017) Molecular mapping of stem rust resistance loci effective against the Ug99 race group of the stem rust pathogen and validation of a single nucleotide polymorphism marker linked to stem rust resistance gene *Sr28*. Phytopathol 107:208-215

Bajgain P, Rouse MN, Bhavani S, Anderson JA (2015) QTL mapping of adult plant resistance to Ug99 stem rust in the spring wheat population RB07/MN06113-8. Mol Breed 35:1-15

Baraibar S, García R, Silva P, Lado B, Castro A, Gutiérrez L, Kavanová M, Quincke M, Bhavani S, Randhawa MS, German S (2020) QTL mapping of resistance to Ug99 and other stem rust pathogen races in bread wheat. Mol Breed 40:1-16

Bokore FE, Knox RE, Cuthbert RD, Pozniak CJ, McCallum BD, N’Diaye A, DePauw RM, Campbell HL, Munro C, Singh A, Hiebert CW, McCartney CA, Sharpe AG, Singh AK, Spaner D, Fowler DB, Ruan Y, Berraies S, Meyer B. (2020) Mapping quantitative trait loci associated with leaf rust resistance in five spring wheat populations using single nucleotide polymorphism markers. PLoS One 15:e0230855

Bokore FE, Cuthbert RD, Hiebert CW, Fetch TG, Pozniak CJ, N’Diaye A, Ruan Y, Meyer B, Knox RE (2021) Mapping stem rust resistance loci effective in Kenya in Canadian spring wheat (Triticum aestivum L.) lines ‘AAC Prevail’and ‘BW961’. Can J Plant Pathol 43:S263-S274

Cheng B, Gao X, Cao N, Ding Y, Chen T, Zhou Q, Gao Y, Xin Z, Zhang L (2022) QTL mapping for adult plant resistance to wheat stripe rust in M96-5× Guixie 3 wheat population.  J Appl Genet 63:265-279

Edae EA, Olivera PD, Jin Y, Poland JA, Rouse MN (2016) Genotype-by-sequencing facilitates genetic mapping of a stem rust resistance locus in *Aegilops umbellulata*, a wild relative of cultivated wheat. BMC Genomics 17:1-10

Gebrewahid TW, Zhang P, Zhou Y, Yan X, Xia X, He Z, Liu D, Li Z (2020) QTL mapping of adult plant resistance to stripe rust and leaf rust in a Fuyu 3/Zhengzhou 5389 wheat population. Crop J 8:655-665

Genievskaya Y, Abugalieva S, Rsaliyev A, Yskakova G, Turuspekov Y (2020) QTL mapping for seedling and adult plant resistance to leaf and stem rusts in Pamyati Azieva× Paragon mapping population of bread wheat. Agronomy 10:1285

Huang S, Wu J, Wang X, Mu J, Xu Z, Zeng Q, Liu S, Wang Q, Kang Z, Han D (2019) Utilization of the genomewide wheat 55K SNP array for genetic analysis of stripe rust resistance in common wheat line P9936. Phytopathol 109:819-827

Huang S, Zhang Y, Ren H, Zhang X, Yu R, Liu S, Zeng Q, Wang Q, Yuan F, Singh RP, Bhavani S, Wu J, Han D, Kang Z (2023) High density mapping of wheat stripe rust resistance gene QYrXN3517-1BL using QTL mapping, BSE-Seq and candidate gene analysis. Theor Appl Genet 136:39

Kolmer JA, Rouse MN (2022) Adult plant leaf rust resistance QTL derived from wheat line CI13227 maps to chromosomes 2AL, 4BS, and 7AL. Plant Genome 15:e20215

Kosgey ZC, Edae EA, Dill-Macky R, Jin Y, Bulbula WD, Gemechu A, Macharia G, Bhavani S, Randhawa MS, Rouse MN (2021) Mapping and validation of stem rust resistance loci in spring wheat line CI 14275. Front Plant Sci 11:609659

Kthiri D, Loladze A, N’Diaye A, Nilsen KT, Walkowiak S, Dreisigacker S, Ammar K, Pozniak CJ (2019) Mapping of genetic loci conferring resistance to leaf rust from three globally resistant durum wheat sources. Front Plant Sci 10:1247

Kumar S, Fetch TG, Knox RE, Singh AK, Clarke JM, Depauw RM, Cuthbert RD, Campbell HL, Singh D, Bhavani S, Pozniak CJ, Meyer B, Clarke FR (2021) Mapping of Ug99 stem rust resistance in Canadian durum wheat. Can J Plant Pathol 43:599-611

Lan C, Basnet BR, Singh RP, Huerta-Espino J, Herrera-Foessel SA, Ren Y, Randhawa MS (2017) Genetic analysis and mapping of adult plant resistance loci to leaf rust in durum wheat cultivar Bairds. Theor Appl Genet 130:609-619

Li Z, Yuan C, Herrera-Foessel SA, Randhawa MS, Huerta-Espino J, Liu D, Dreisigacker S, Singh RP, Lan C (2020) Four consistent loci confer adult plant resistance to leaf rust in the durum Wheat Lines Heller# 1 and Dunkler. Phytopathol 110:892-899

Liu L, Wang M, Feng J, See D, Chao S, Chen X (2018) Combination of all-stage and high-temperature adult-plant resistance QTL confers high-level, durable resistance to stripe rust in winter wheat cultivar Madsen. Theor Appl Genet 131:1835-1849

Marone D, Mazzucotelli E, Matny O, Desiderio F, Sciara G, Maccaferri M, Marcotuli I, Gadaleta A, Steffenson B, Mastrangelo AM (2022) QTL Mapping of Stem Rust Resistance in Populations of Durum Wheat. Genes 13:1793

Megerssa SH, Ammar K, Acevedo M, Bergstrom GC, Dreisigacker S, Randhawa M, Brown-Guedira G, Ward B, Sorrells ME (2022) QTL mapping of seedling and field resistance to stem rust in DAKIYE/Reichenbachii durum wheat population. PLoS One 17:e0273993

Mu J, Huang S, Liu S, Zeng Q, Dai M, Wang Q, Wu J, Yu S, Kang Z, Han D (2019a) Genetic architecture of wheat stripe rust resistance revealed by combining QTL mapping using SNP-based genetic maps and bulked segregant analysis. Theor Appl Genet 132:443-455

Mu J, Wu J, Liu S, Dai M, Sun D, Huang S, Wang Q, Zeng Q, Yu S, Chen L, Kang Z, Han D (2019b) Genome-wide linkage mapping reveals stripe rust resistance in common wheat (*Triticum aestivum*) Xinong1376. Plant Dis 103:2742-2750

Pujol V, Forrest KL, Zhang P, Rouse MN, Hayden MJ, Huang L, Tabe L, Lagudah E (2015) Identification of a stem rust resistance locus effective against Ug99 on wheat chromosome 7AL using a RAD-Seq approach. Theor Appl Genet 128:1397-1405

Rauf Y, Bajgain P, Rouse MN, Khanzada KA, Bhavani S, Huerta-Espino J, Singh RP, Imtiaz M, Anderson JA (2022) Molecular characterization of genomic regions for adult plant resistance to stem rust in a spring wheat mapping population. Plant Dis 106:439-450

Rosa SB, Zanella CM, Hiebert CW, Brûlé-Babel AL, Randhawa HS, Shorter S, Boyd LA, McCallum BD (2019) Genetic characterization of leaf and stripe rust resistance in the Brazilian wheat cultivar Toropi. Phytopathol 109:1760-1768

Wu J, Wang Q, Liu S, Huang S, Mu J, Zeng Q, Huang L, Han D, Kang Z (2017) Saturation mapping of a major effect QTL for stripe rust resistance on wheat chromosome 2B in cultivar Napo 63 using SNP genotyping arrays. Front Plant Sci 8:653

Wu J, Liu S, Wang Q, Zeng Q, Mu J, Huang S, Yu S, Han D, Kang Z (2018) Rapid identification of an adult plant stripe rust resistance gene in hexaploid wheat by high-throughput SNP array genotyping of pooled extremes. Theor Appl Genet 131:43-58

Xu X, Li G, Bai G, Bernardo A, Carver BF, St. Amand P, Bian R (2021) Characterization of an incomplete leaf rust resistance gene on chromosome 1RS and development of KASP markers for *Lr47* in wheat. Phytopathol 111:649-658

Yang Y, Basnet BR, Ibrahim AM, Rudd JC, Chen X, Bowden RL, Xue Q, Wang S, Johnson CD, Metz R, Mason RE, Hays DB, Liu S (2019) Developing KASP markers on a major stripe rust resistance QTL in a popular wheat TAM 111 using 90K array and genotyping‐by‐sequencing SNPs. Crop Sci 59:165-175

Zeng Q, Wu J, Huang S, Yuan F, Liu S, Wang Q, Mu J, Yu S, Chen L, Han D, Kang Z (2019a) SNP-based linkage mapping for validation of adult plant stripe rust resistance QTL in common wheat cultivar Chakwal 86. Crop J 7:176-186

Zeng Q, Wu J, Liu S, Chen X, Yuan F, Su P, Wang Q, Huang S, Mu J, Han D, Kang Z, Chen XM (2019b) Genome-wide mapping for stripe rust resistance loci in common wheat cultivar Qinnong 142. Plant Dis 103:439-447

Zhang P, Yin G, Zhou Y, Qi A, Gao F, Xia X, He Z, Li Z, Liu D (2017) QTL mapping of adult-plant resistance to leaf rust in the wheat cross Zhou 8425B/Chinese Spring using high-density SNP markers. Front Plant Sci 8:793

Zhang P, Li X, Gebrewahid TW, Liu H, Xia X, He Z, Li Z, Liu D (2019) QTL mapping of adult-plant resistance to leaf and stripe rust in wheat cross SW 8588/Thatcher using the wheat 55K SNP array. Plant Dis 103:3041-3049

Zhang P, Lan C, Singh RP, Huerta-Espino J, Li Z, Lagudah E, Bhavani S (2022) Identification and characterization of resistance loci to wheat leaf rust and stripe rust in Afghan landrace “KU3067”. Front Plant Sci 13:894528

Zurn JD, Rouse MN, Chao S, Aoun M, Macharia G, Hiebert CW, Pretorius ZA, Bonman JM, Acevedo M (2018) Dissection of the multigenic wheat stem rust resistance present in the Montenegrin spring wheat accession PI 362698. BMC Genomics 19:1-11
