# Supplementary material for: Wheat improvement through advances in single nucleotide polymorphism (SNP) detection and genotyping with a special emphasis on rust resistance
Source: Theor Appl Genet. 2024 Sep 16;137(10):224. doi: 10.1007/s00122-024-04730-w (PMC11405505; doi:10.1007/s00122-024-04730-w)
Supplement: Supplementary file 5 — Supplementary file5 (DOCX 38 KB) [file 122_2024_4730_MOESM5_ESM.docx]

**Supplementary table 3: SNP-based detection of novel QTLs for rust resistance through genome-wide association analysis (GWAS)**

| Disease | Accessions | Resistance stage | Genotyping platform | No. of SNPs surveyed | No. of QTLs/MTAs/candidate SNPs* | Chromosomes | Reference |
| --- | --- | --- | --- | --- | --- | --- | --- |
| Leaf rust | 496 *T. durum* wheat accessions | ASR, APR | 9K SNP array | 3569 | 88(14) | All chromosomes except for 1B and 7B | Aoun et al. (2016) |
| Leaf rust | 338 spring wheat breeding lines | ASR, APR | 90K SNP array | 18925 | 46(2) | 1A,7B,4B, 5B, 4A, 5D, 6B | Gao et al. (2016) |
| Leaf rust | 159 landraces and old cultivars | ASR, APR | 9K SNP array | 670 | 23(1) | 1B, 1D, 3B, 4A, 5B, 6A, 6B, 6D, 7A | Kankwatsa et al. (2017) |
| Leaf rust | 676 pre-green revolution common wheat landrace accessions collected in the 1920–1930s by A.E. Watkins | APR | 90K SNP array | 15458 | 5(3) | 1B, 2B, 5A | Pasam et al. (2017) |
| Leaf rust | 1032 accessions from USDA National Small Grains Core Collection | ASR, APR | 9K SNP array  KASP | 5729 | 52(15) | 2BL, 4AS, 5DL, 2DL, 7AS | Turner et al. (2017) |
| Leaf rust | 96 winter wheat accessions including cultivars and advanced breeding lines | APR | DArT | 3874 | 13(6) | 1BL, 1DS, 2AS, 2BL, 2DS, 3BS, 3BL, 4AL, 6BS, 7DS, 5BL/7BS and 6AL/6BS | Gerard et al. (2018) |
| Leaf rust | 333 and 313 lines from 45^th^ and 46^th^ International Bread Wheat Screening Nursery (IBWSN) respectively | ASR | GBS | 3510 + 8072 | 12 | 1DS, 2AS, 2BL, 3B, 4AL, 6AS and 6AL | Juliana et al. (2018) |
| Leaf rust | 125 synthetic hexaploid wheat accessions | APR | GBS | 35798 | 46 | 1A, 1B, 1D 2A, 2B, 2D, 3A, 3B, 4A, 4B, 4D, 5A, 5B, 5D, 6A, 6B, 7A, 7B | Bhatta et al. (2019) |
| Leaf rust | CIMMYT’s elite yield trial panel of 3,485 lines | ASR, APR | GBS | 77,148 | 2 | 1D, 2B | Juliana et al. (2019) |
| Leaf rust | 331 accessions consisting of 297 spring wheat and 34 soft red winter wheat types | ASR | 9K SNP array | 5025 | 11(5) | 1AL, 1BL, 2BS, 4AS, 4BL,5AS, 6AL 7AS, 7BL | Sapkota et al. (2019) |
| Leaf rust | 385 accessions, including 27 different species of cultivated wheat, SHWs, progenitor species and wild relatives | APR | 90K SNP array Exome capture | 9570 | 96(75) | All chromosomes | Fatima et al. (2020) |
| Leaf rust | 483 spring wheat accessions | ASR, APR | 35K SNP array | 14650 | 18 | 1A, 1B, 2B, 3A, 3B, 3D, 5B, 7A | Kumar et al. (2020) |
| Leaf rust | 100 Russian spring wheat varieties | APR | 15K SNP array | 9406 | 20(2) | 6D, 6A, 6B, 5A, 1B, 2A, 2B, 7A | Leonova et al. (2020) |
| Leaf rust | 268 lines including 208 Chinese accessions | ASR | 90K SNP array | 12931 | 22(18) | Distributed on 14 chromosomes | Zhang et al. (2021) |
|  |  | APR |  |  | 7(3) | 1BL, 2AL.1, 2BS, 3BS, 4AL, 6BL, 7BS |  |
| Leaf rust | 193 tetraploid wheat accessions | ASR, APR | 90K SNP array | 16425 | 17(6) | 2A, 2B, 3A, 3B, 6A, 6B, 7A, 7B | Genievskaya et al. (2022) |
| Leaf rust | 196 spring wheat accessions | APR | 90K SNP array KASP | 23342 | 13 | 1A, 2A, 2B, 2D, 3A, 3B, 3D, 5A, 5B, 7B | Iqbal et al. (2022) |
| Leaf rust | 2300 worldwide wheat accessions | APR | 90K SNP array | 9526 | 13(4) | All chromsomes except 1D, 4B, 5A, 5D, 6D, 7A | Joukhadar et al. (2022) |
| Leaf rust | 198 spring wheat accessions | ASR | GBS  9K SNP array | 11362  6883 | 3  2 | 6D  6A, 6B | Mourad et al. (2022) |
| Leaf rust | 168 pre-breeding lines | APR | GBS DART | 58378 | 57 | 1B, 1D, 2A, 2B, 2D, 3A, 3B, 3D, 4A, 4D, 5A, 5B, 6A, 6B, 6D, 7A, 7B,7D | Saleem et al. (2022) |
| Leaf rust | 400 bread wheat accessions | ASR | GBS | 18932 | 51 | 19 wheat chromosomes  1A, 1B, 1D, 2B, 2D, 3D, 5B, 6B, 6D, 7B, 7D, 3A, 3B,7A, 2A | Vikas et al. (2022) |
|  |  | APR |  |  | 15(5) | 1A, 1B, 1D, 2A, 2B, 3A, 3B, 3D, 4A, 4D, 6D |  |
| Leaf rust | 212 local bread wheat accessions | ASR, APR | 20K SNP array | 11150 | 11 | 1A, 1B, 1D, 2A, 4B, 5B, 6A, 7A | Zatybekov et al. (2022) |
| Leaf rust | 320 Iranian bread wheat cultivars and landraces | ASR  APR | GBS | 46203 | 80(6) | All chromosomes, except for 1D, 3D, 4D, and 7D | Delfan et al. (2023) |
| Leaf rust | 447 accessions of three *T. turgidum* subspecies | ASR | 90K SNP array | 6412 | 1 | 1A | Klymiuk et al. (2023) |
| Leaf rust | 180 cultivated emmer accessions from USDA-ARS National Small Grains Collection | ASR | GBS  9K SNP array | 46383  4331 | 15(13) | 2B, 6B, 2A, 3B, 4A, 5A, 7A, 7B | Lhamo et al. (2023) |
|  |  | APR |  |  | 11(10) | 2B, 3B, 6A 1A, 4A, 5B, 6B |  |
| Stripe rust | 1175 accessions in the National Small Grains Collection winter wheat germplasm collection | ASR,  APR | 9K SNP array | 5347 | 127 (5) | 1A, 1B, 2A, 3A, 4A, 4D, 5A, 5B,6A, 6B, | Bulli et al. (2016) |
| Stripe rust | 153 bread-wheat landraces | APR | GBS DART | 64000 | 13(6) | 1B, 2A, 2D, 3B, 4B, 5B, 6B | Sehgal et al. (2016) |
| Stripe rust | 124 landraces, 66 commercial cultivars and breeding lines from the Ethiopian Institute of Agricultural Research (EIAR) | ASR, APR | 90K SNP array | 24281 | 15(3) | 1B, 2A, 4A, 5A, 6A, 7B | Muleta et al. (2017) |
| Stripe rust | 676 pre-green revolution common wheat landrace accessions collected in the 1920–1930s by A.E. Watkins | APR | 90K SNP array | 15458 | 14(12) | 1A, 1B, 3A, 5B, 5D, 6A, 7A, 7B | Pasam et al. (2017) |
| Stripe rust | 333 and 313 lines from 45^th^ and 46^th^ International Bread Wheat Screening Nursery (IBWSN) respectively | ASR | GBS | 3510 + 8072 | 10 | 2AS, 2DL, 3B, 7DS | Juliana et al. (2018) |
| Stripe rust | 125 synthetic hexaploid wheat accessions | APR | GBS | 35798 | 11 | 1A, 2A, 3B, 3D, 6A, 6B, 7A, 7B, 7D | Bhatta et al. (2019) |
| Stripe rust | EYT panel of 3,485 lines | APR | GBS | 77,148 | 4 | 2A, 2B, 3A | Juliana et al. (2019) |
| Stripe rust | 328 *T. durum* lines | APR | GBS DArTSeq | 12550 | 6 | 3A,4B, 1B, 2B | Miedaner et al. (2019) |
| Stripe rust | 158 wheat cultivars | ASR | GBS DArTSeq | 21,543 | 13 | 1A, 1B, 2A, 2B, 3A, 3B, 6A, 6B, 7B, 7D | Miedaner et al. (2020) |
| Stripe rust | 483 spring wheat accessions | ASR, APR | 35KSNP array | 14650 | 16 | 1A, 1B, 1D, 2A, 2B, 3B, 5A, 6A | Kumar et al. (2020) |
| Stripe rust | 103 spring wheat genotypes | APR | GBS | 26,703 | 5 | 2A,4A | Abou-zeid and Mourad (2021) |
| Stripe rust | 141 advanced wheat breeding lines | APR | GBS | 14563 | 8(4) | 2A, 2D, 5B, 6A, 7A | Tomar et al. (2021) |
| Stripe rust | 411 spring wheat lines | APR | 660K SNP array | 371 972 | 19(5) | 1AL, 1BL, 2AS, 2AL, 2BS, 2BL, 3AL, 3BS, 3BL, 4BS, 4BL, 6BS, 6BL, 7AL, 7BL, 7DS | Wu et al. (2021) |
| Stripe rust | 268 lines including 208 Chinese accessions | APR | 90K SNP array | 12931 | 6(2) | 1Bl,1DS, 2AL, 6BS, 7BL | Zhang et al. (2021) |
| Stripe rust | 196 spring wheat types | APR | 90K SNP array KASP | 23342 | 5 | 1B,2B, 4B,5A,7D | Iqbal et al. (2022) |
| Stripe rust | 2300 worldwide wheat accessions | APR | 90K SNP array | 9526 | 57(7) | All chromosomes except 1D, 2D, 5D, 6A and 6D | Joukhadar et al. (2022) |
| Stripe rust | 168 pre-breeding lines | APR | GBS DART | 58378 | 36 | 1A, 1B, 1D, 2A, 2B, 2D, 3B, 4D, 5A, 5B, 5D, 6A, 7A, 7B | Saleem et al. (2022) |
| Stripe rust | 419 wild emmer wheat (WEW) accessions  188 WEW accessions | ASR  APR | GBS  GBS | 202,113  44,052 | 2  13(3) | 1BS, 3AS, 1A,1B, 3B, 5B, 6B,7A,7B | Tene et al. (2022) |
| Stripe rust | 447 accessions of three *T. turgidum* subspecies | ASR | 90K SNP array | 6412 | 2 | 1B, 7B | Klymiuk et al. (2023) |
| Stem rust | 10 RIL populations (852 lines) involving stem rust susceptible line ‘LMPG-6’ as a common parent | APR | GBS | 13413 SNPs | 14 | 20 out of 21 chromosomes | Bajgain et al. (2016) |
| Stem rust | 2152 spring wheat accessions | ASR | 9K SNP array | 6223 SNPs | 47 (10) | 1A , 2B, 2D, 3B, 4A, 6A | Gao et al. (2017) |
| Stem rust | 159 landraces and old cultivars | ASR, APR | 9K SNP array | 670 | 41 | 1B, 1D, 2A, 2B, 3A, 3B, 4A, 5A, 5B, 6A, 6B, 7A, 7B | Kankwatsa et al. (2017) |
| Stem rust | 1411 hexaploid winter wheat accessions | ASR, APR | 9K SNP array | 5390 | 10(3) | 1A, 1BL. RS, 2A, 2B, 2D, 6A | Mihalyov et al. (2017) |
| Stem rust | 124 landraces, 66 commercial cultivars and breeding lines from the Ethiopian Institute of Agricultural Research | ASR | 90K SNP array | 24281 | 9(2) | 1B, 2A, 2B, 3B, 4A, 6A, 6B, 7B | Muleta et al. (2017) |
| Stem rust | 676 pre-green revolution common wheat landrace accessions collected in the 1920–1930s by A.E. Watkins | APR | 90K SNP array | 15458 | 11 (9) | 3A, 3B, 4B, 5A, 6A, 7B | Pasam et al. (2017) |
| Stem rust | 230 tetraploid wheat accessions | ASR | 90K SNP array | 17678 | 22(6) | 1B, 5A, 5B, 6B, 7B | Saccomanno et al. (2018) |
| Stem rust | 125 synthetic hexaploid wheat accessions | APR | GBS | 35798 | 52 | 1A, 1B, 1D 2A, 2D, 3A, 3B, 4A, 4B, 4D, 5A, 5B, 5D, 6A, 6B, 6D,7A, 7B, 7D | Bhatta et al. (2019) |
| Stem rust | EYT panel of 3,485 lines | ASR, APR | GBS | 77,148 | 11 | 2A, 3A, 3D, 6B, 3B, 6AS, 7D | Juliana et al. (2019) |
| Stem rust | 328 *T. durum* lines | APR | GBS DArTSeq | 12550 | 6 | 6AS, 5B, 1A, 3A | Miedaner et al. (2019) |
| Stem rust | 158 cultivars | ASR | GBS  DArTSeq | 21,543 | 8 | 1B, 2A, 2A, 3D, 4A, 4D, 7A, 7D | Miedaner et al. (2020) |
| Stem rust | 250 North American spring wheat lines | ASR | GBS | 9042 | 23 | 1B , 4AL, 6AS, 6BL | Edae and Rouse (2020) |
| Stem rust | 483 spring wheat accessions | APR, ASR | 35K SNP array | 14650 | 27 | 1A , 1B, 1D , 2A , 2B, 3A, 3B , 3D, 5A, 5B, 6B, 6D, 7A | Kumar et al. (2020) |
| Stem rust | 283 *T. durum* lines assembled by the International Maize and Wheat Improvement Center | APR | GBS | 26,439 | 42(21) | 1A,1B, 2A, 2B, 3A, 3B, 4A, 5A, 5B, 6A, 6B, 7A, 7B | Megersa et al. (2020) |
| Stem rust | 212 randomly selected genotypes from 270 F_3:6_ lines (Nebraska Duplicate Nursery, syn. DUP 2017) | ASR | GBS | 11911 | 84 | 1B, 2A, 2B, 7B and an unknown chromosome | Elather et al. (2021) |
| Stem rust | 193 tetraploid wheat accessions | ASR, APR | 90K SNP array | 16425 | 21(5) | 1A,1B,2A,2B,3A,3B,5A,5B,6A,6B,7A | Genievskaya et al. (2022) |
| Stem rust | 2300 worldwide wheat accessions | APR | 90K SNP array | 9526 | 18(5) | All chromosomes except 1A, 2A, 4A, 4D, 6D | Joukhadar et al. (2020) |
| Stem rust | 245 elite lines from ICARDA | APR | 15K SNP array | 9523 | 11(7) | 1B, 3A, 3B, 4A, 4B, 5A | Shewabez et al. (2022) |
| Stem rust | 212 local bread wheat accessions | APR, ASR | 20K SNP array | 11150 | 8 | 1B, 1D, 2A, 4B, 6A, 7A | Zatybekov et al. (2021) |
| Stem rust | 447 accessions of three *T. turgidum* subspecies | ASR | 90K SNP array | 6412 | 2 | 6A, 2A | Klymiuk et al. (2023) |

* Values in parenthesis indicate novel QTLs out of the total QTLs/ marker-trait associations identified

**References**

Abou-Zeid MA, Mourad AMI (2021) Genomic regions associated with stripe rust resistance against the Egyptian race revealed by genome-wide association study. BMC Plant Biol 21:1-14

Aoun M, Breiland M, Kathryn Turner M, Loladze A, Chao S, Xu SS, Ammar K, Anderson JA, Kolmer JA, Acevedo M (2016) Genome‐wide association mapping of leaf rust response in a durum wheat worldwide germplasm collection. Plant Genome 9:3835

Bajgain P, Rouse MN, Tsilo TJ, Macharia GK, Bhavani S, Jin Y, Anderson JA (2016) Nested association mapping of stem rust resistance in wheat using genotyping by sequencing. PloS One 11:e0155760

Bhatta M, Shamanin V, Shepelev S, Baenziger PS, Pozherukova V, Pototskaya I, Morgounov A (2019) Marker-trait associations for enhancing agronomic performance, disease resistance, and grain quality in synthetic and bread wheat accessions in Western Siberia. Genes Genom Genet 9:4209-4222

Bulli P, Zhang J, Chao S, Chen X, Pumphrey M (2016) Genetic architecture of resistance to stripe rust in a global winter wheat germplasm collection. Genes Genom Genet 6:2237-2253

Delfan S, Bihamta MR, Dadrezaei ST, Abbasi A, Alipour H (2023) Exploring genomic regions involved in bread wheat resistance to leaf rust at seedling/adult stages by using GWAS analysis. BMC Genomics 24:83

Edae EA, Rouse MN (2020) Association mapping of resistance to emerging stem rust pathogen races in spring wheat using genotyping‐by‐sequencing. Plant Genome 13:e20050

Eltaher S, Mourad AM, Baenziger PS, Wegulo S, Belamkar V, Sallam A (2021) Identification and validation of high LD hotspot genomic regions harboring stem rust resistant genes on 1B, 2A (*Sr38*), and 7B chromosomes in wheat. Front Genet 12:749675

Fatima F, McCallum BD, Pozniak CJ, Hiebert CW, McCartney CA, Fedak G, You FM, Cloutier S (2020) Identification of new leaf rust resistance loci in wheat and wild relatives by array-based SNP genotyping and association genetics. Front Plant Sci 11:583738

Gao L, Turner MK, Chao S, Kolmer J, Anderson JA (2016) Genome wide association study of seedling and adult plant leaf rust resistance in elite spring wheat breeding lines. PLoS One 11:e0148671

Gao L, Rouse MN, Mihalyov PD, Bulli P, Pumphrey MO, Anderson JA (2017) Genetic characterization of stem rust resistance in a global spring wheat germplasm collection. Crop Sci 57:2575-2589

Genievskaya Y, Pecchioni N, Laidò G, Anuarbek S, Rsaliyev A, Chudinov V, Zatybekov A, Turuspekov Y, Abugalieva S (2022) Genome-wide association study of leaf rust and stem rust seedling and adult resistances in tetraploid wheat accessions harvested in kazakhstan. Plants 11:1904

Gerard GS, Kobiljski B, Lohwasser U, Börner A, Simon MR (2018) Genetic architecture of adult plant resistance to leaf rust in a wheat association mapping panel. Plant Pathol 67:584-594

Iqbal M, Semagn K, Jarquin D, Randhawa H, McCallum BD, Howard R, Aboukhaddour R, Ciechanowska I, Strenzke K, Crossa J, Céron-Rojas JJ, N'Diaye A, Pozniak C, Spaner D (2022) Identification of disease resistance parents and genome-wide association mapping of resistance in spring wheat. Plants 11:2905

Joukhadar R, Hollaway G, Shi F, Kant S, Forrest K, Wong D, Petkowski J, Pasam R, Tibbits J, Bariana H, Bansal U, Spangenberg G, Daetwyler H, Gendall T, Hayden M (2020) Genome-wide association reveals a complex architecture for rust resistance in 2300 worldwide bread wheat accessions screened under various Australian conditions. Theor Appl Genet 133:2695-2712

Juliana P, Singh RP, Singh PK, Poland JA, Bergstrom GC, Huerta-Espino J, Bhavani S, Crossa J, Sorrells ME (2018) Genome-wide association mapping for resistance to leaf rust, stripe rust and tan spot in wheat reveals potential candidate genes. Theor Appl Genet 131:1405-1422

Juliana P, Poland J, Huerta-Espino J, Shrestha S, Crossa J, Crespo-Herrera L, Toledo FH, Govindan V, Mondal S, Kumar U, Bhavani S, Singh PK, Randhawa MS, He X, Guzman C, Dreisigacker S, Rouse MN, Jin Y, Pérez-Rodríguez P, Montesinos-López OA, Singh D, Mokhlesur Rahman M, Marza F, Singh RP (2019) Improving grain yield, stress resilience and quality of bread wheat using large-scale genomics. Nat Genet 51:1530-1539

Kankwatsa P, Singh D, Thomson PC, Babiker EM, Bonman JM, Newcomb M, Park RF (2017) Characterization and genome-wide association mapping of resistance to leaf rust, stem rust and stripe rust in a geographically diverse collection of spring wheat landraces. Mol Breed 37:1-24

Klymiuk V, Haile T, Ens J, Wiebe K, N’Diaye A, Fatiukha A, Krugman T, Ben-David R, Hübner S, Cloutier S, Pozniak CJ (2023) Genetic architecture of rust resistance in a wheat (*Triticum turgidum*) diversity panel. Front Plant Sci 14:1145371

Kumar D, Kumar A, Chhokar V, Gangwar OP, Bhardwaj SC, Sivasamy M, Prasad SS, Prakasha T, Khan H, Singh R, Sharma P, Sheoran S, Iquebal MA, Jaiswal S, Angadi UB, Singh G, Rai A, Singh GP, Kumar D, Tiwari R (2020) Genome-wide association studies in diverse spring wheat panel for stripe, stem, and leaf rust resistance. Front Plant Sci 11:748

Leonova IN, Skolotneva ES, Salina EA (2020) Genome-wide association study of leaf rust resistance in Russian spring wheat varieties. BMC Plant Biol 20:1-13

Lhamo D, Sun Q, Zhang Q, Li X, Fiedler JD, Xia G, Faris JD, Gu Y-Q, Gill U, Cai X, Acevedo M, Xu SS (2023) Genome-wide association analyses of leaf rust resistance in cultivated emmer wheat. Theor Appl Genet 136:20

Megerssa SH, Ammar K, Acevedo M, Brown-Guedira G, Ward B, Degete AG, Randhawa MS, Sorrells ME (2020) Multiple-race stem rust resistance loci identified in durum wheat using genome-wide association mapping. Front Plant Sci 11:598509

Miedaner T, Rapp M, Flath K, Longin CFH, Würschum T (2019) Genetic architecture of yellow and stem rust resistance in a durum wheat diversity panel. Euphytica 215:1-17Miedaner T, Akel W, Flath K, Jacobi A, Taylor M, Longin F, Würschum T (2020) Molecular tracking of multiple disease resistance in a winter wheat diversity panel. Theor Appl Genet 133:419-431

Mihalyov PD, Nichols VA, Bulli P, Rouse MN, Pumphrey MO (2017) Multi‐locus mixed model analysis of stem rust resistance in winter wheat. Plant Genome <https://doi.org/10.3835/plantgenome2017.01.0001>

Mourad AM, Draz IS, Omar GE, Börner A, Esmail SM (2022) Genome-wide screening of broad-spectrum resistance to leaf rust (*Puccinia triticina* Eriks) in Spring wheat (*Triticum aestivum* L.). Front Plant Sci 13:921230

Muleta KT, Rouse MN, Rynearson S, Chen X, Buta BG, Pumphrey MO (2017) Characterization of molecular diversity and genome-wide mapping of loci associated with resistance to stripe rust and stem rust in Ethiopian bread wheat accessions. BMC Plant Biol 17:1-20

Pasam RK, Bansal U, Daetwyler HD, Forrest KL, Wong D, Petkowski J, Willey N, Randhawa M, Chhetri M, Miah H, Tibbits J, Bariana H, Hayden MJ (2017) Detection and validation of genomic regions associated with resistance to rust diseases in a worldwide hexaploid wheat landrace collection using BayesR and mixed linear model approaches. Theor Appl Genet 130:777-793

Saccomanno A, Matny O, Marone D, Laidò G, Petruzzino G, Mazzucotelli E, Desiderio F, Blanco A, Gadaleta A, Pecchioni N, De Vita P, Steffenson B, Mastrangelo AM (2018) Genetic mapping of loci for resistance to stem rust in a tetraploid wheat collection. Int J Mol Sci 19:3907

Saleem K, Shokat S, Waheed MQ, Arshad HMI, Arif MAR (2022) A GBS-based GWAS analysis of leaf and stripe rust resistance in diverse pre-breeding germplasm of bread wheat (*Triticum aestivum* L.). Plants 11:2363

Sapkota S, Hao Y, Johnson J, Buck J, Aoun M, Mergoum M (2019) Genome‐wide association study of a worldwide collection of wheat genotypes reveals novel quantitative trait loci for leaf rust resistance. Plant Genome 12:190033

Sehgal D, Dreisigacker S, Belen S, Küçüközdemir Ü, Mert Z, Özer E, Morgounov A (2016) Mining centuries old in situ conserved Turkish wheat landraces for grain yield and stripe rust resistance genes. Front Genet 7:201

Shewabez E, Bekele E, Alemu A, Mugnai L, Tadesse W (2022) Genetic characterization and genome-wide association mapping for stem rust resistance in spring bread wheat. BMC Genom Data 23:1-15

Tene M, Adhikari E, Cobo N, Jordan KW, Matny O, del Blanco IA, Roter J, Ezrati S, Govta L, Manisterski J, Yehuda PB, Chen X, Steffenson B, Akhunov E, Sela H (2022) GWAS for stripe rust resistance in wild emmer wheat (*Triticum dicoccoides*) population: obstacles and solutions. Crops 2:42-61

Tomar V, Dhillon GS, Singh D, Singh RP, Poland J, Chaudhary AA, Bhati PK, Joshi AK, Kumar U (2021) Evaluations of genomic prediction and identification of new loci for resistance to stripe rust disease in wheat (*Triticum aestivum* L.). Front Genet 12:710485

Turner MK, Kolmer JA, Pumphrey MO, Bulli P, Chao S, Anderson JA (2017) Association mapping of leaf rust resistance loci in a spring wheat core collection. Theor Appl Genet 130:345-361

Vikas V, Pradhan AK, Budhlakoti N, Mishra DC, Chandra T, Bhardwaj S, Kumar S, Sivasamy M, Jayaprakash P, Nisha R, Shajitha P, Peter J, Geetha M, Mir RR, Singh K, Kumar S (2022) Multi-locus genome-wide association studies (ML-GWAS) reveal novel genomic regions associated with seedling and adult plant stage leaf rust resistance in bread wheat (*Triticum aestivum* L.). Heredity 128:434-449

Wu J, Yu R, Wang H, Zhou Ce, Huang S, Jiao H, Yu S, Nie X, Wang Q, Liu S, Weining S, Singh RP, Bhavani S, Kang Z, Han D, Zeng Q (2021) A large‐scale genomic association analysis identifies the candidate causal genes conferring stripe rust resistance under multiple field environments. Plant Biotechnol J 19:177-191

Zatybekov A, Genievskaya Y, Rsaliyev A, Maulenbay A, Yskakova G, Savin T, Turuspekov Y, Abugalieva S (2021) Identification of quantitative trait loci for leaf rust and stem rust seedling resistance in bread wheat using a genome-wide association study. Plants 11:74

Zhang P, Yan X, Gebrewahid TW, Zhou Y, Yang E, Xia X, He Z, Li Z, Liu D (2021) Genome-wide association mapping of leaf rust and stripe rust resistance in wheat accessions using the 90K SNP array. Theor Appl Genet 134:1233-1251
